# The Two Faces of Janus: Why Thyrotropin as a Cardiovascular Risk Factor May Be an Ambiguous Target

**DOI:** 10.3389/fendo.2020.542710

**Published:** 2020-10-26

**Authors:** Johannes Wolfgang Dietrich, Rudolf Hoermann, John E. M. Midgley, Friederike Bergen, Patrick Müller

**Affiliations:** ^1^ Endocrinology and Diabetes Department, Medical Hospital I, Bergmannsheil University Hospitals, Ruhr University of Bochum, Bochum, Germany; ^2^ Ruhr Center for Rare Diseases (CeSER), Ruhr University of Bochum and Witten/Herdecke University, Bochum, Germany; ^3^ Private Consultancy, Research and Development, Yandina, QLD, Australia; ^4^ North Lakes Clinical, Ilkley, United Kingdom; ^5^ Department of Psychiatry and Psychotherapy, LVR-Klinikum Düsseldorf, Düsseldorf, Germany; ^6^ Department of Cardiology II, Münster University Hospitals, University of Münster, Münster, Germany

**Keywords:** allostatic load, subclinical hypothyroidism, thyroid homeostasis, thyrotropin, malignant arrhythmia, sudden cardiac death, post-traumatic stress disorder (PTSD)

## Abstract

Elevated concentrations of free thyroid hormones are established cardiovascular risk factors, but the association of thyrotropin (TSH) levels to hard endpoints is less clear. This may, at least in part, ensue from the fact that TSH secretion depends not only on the supply with thyroid hormones but on multiple confounders including genetic traits, medication and allostatic load. Especially psychosocial stress is a still underappreciated factor that is able to adjust the set point of thyroid function. In order to improve our understanding of thyroid allostasis, we undertook a systematic meta-analysis of published studies on thyroid function in post-traumatic stress disorder (PTSD). Studies were identified *via* MEDLINE/PubMed search and available references, and eligible were reports that included TSH or free thyroid hormone measurements in subjects with and without PTSD. Additionally, we re-analyzed data from the NHANES 2007/2008 cohort for a potential correlation of allostatic load and thyroid homeostasis. The available evidence from 13 included studies and 3386 euthyroid subjects supports a strong association of both PTSD and allostatic load to markers of thyroid function. Therefore, psychosocial stress may contribute to cardiovascular risk *via* an increased set point of thyroid homeostasis, so that TSH concentrations may be increased for reasons other than subclinical hypothyroidism. This provides a strong perspective for a previously understudied psychoendocrine axis, and future studies should address this connection by incorporating indices of allostatic load, peripheral thyroid hormones and calculated parameters of thyroid homeostasis.

## Introduction

The prognostic and therapeutic implications of subclinical hypothyroidism remain to be debated (1–4). At least in elderly subjects, the benefits of levothyroxine substitution are questionable (5). While increased and high-normal free thyroxine (FT4) concentration is a well-established risk factor for malignant arrhythmia and sudden cardiac death (6, 7), the association between thyrotropin (TSH) concentration and cardiovascular mortality is less well understood (8–11). Studies reported either no relation at all (12, 13) or a rather complex U-shaped association (14, 15), as has been shown in a recent population study based on the large NHANES datasets (16). Likewise, a recent study observed elevated FT4 concentration in stress cardiomyopathy or Takotsubo syndrome (17). In the same cohort, the distribution of TSH levels was complex and ambiguous, however.

The conclusion by Inoue et al. that subclinical hypothyroidism is a risk factor for cardiovascular death (16) may be premature. High-normal or elevated TSH concentrations do not unequivocally indicate early thyroid failure but may also be reflective of an increased homeostatic set point of the hypothalamus-pituitary-thyroid axis (18). Apart from genetic traits manifestation of a high set point may ensue from chronic psychosocial stress. This is termed type 2 allostatic load and a well-recognized cardiovascular risk indicator (19–24). Set point alterations have been linked to acute and chronic stress situations including psychosis, alcohol withdrawal and post-traumatic stress disorder (PTSD), all of which are known risk factors for cardiovascular mortality (25–30). Here, we present a brief evaluation of the stress-thyroid axis and discuss an alternative explanation.

## Methods

Based on previous reports on a possible association of PTSD to thyroid function (19, 31–36) we performed a systematic meta-analysis summarizing the available evidence. Up to July 4^th^, 2020, a systematic search was executed of PubMed/MEDLINE by using the following search formula: “post-traumatic stress disorder AND (thyroid OR triiodothyronine OR thyrotropin OR TSH OR thyroxine)”. Additionally, we screened the references of the retrieved publications for additional suitable publications. Studies were eligible if they compared TSH, FT4 or free T3 (FT3) concentration in subjects with and without PTSD, and if the definition of the disease was compatible with the criteria provided by the fifth edition of the Diagnostic and Statistical Manual of Mental Disorders (DSM-5). Summary measures were included in random effects meta-analysis. Between-study variance was assessed with the DerSimonian-Laird estimator, Cochran’s Q and tau squared, and heterogeneity with Higgins’ and Thompson’s I squared. Small study effects were estimated with funnel plots and three tests statistics (Egger, Begg and Mazumdar, and Thompson and Sharp) with the null hypothesis of no bias in the meta-analysis. The quality of the studies was assessed with a Newcastle-Ottawa score. Calculations were supported by the packages meta and metaphor (37, 38) for the statistical environment R (39).

Additionally, we analyzed publicly available data from the NHANES 2007/2008 dataset (40) in order to re-assess the association between thyroid function and type 2 allostasis. A summative quantitative allostatic load score (SIQALS 2) was derived from the sum of sub-scores for pulse rate, systolic and diastolic blood pressure, total cholesterol, high density lipoprotein (HDL), body mass index (BMI), HbA1c and c-reactive protein (CRP) concentration according to the procedure of the Scottish Health Survey (20). Each sub-score was graded as 1 if the results were in the upper quartile of the respective population distribution or 0 if they were in any of the lower quartiles, except for HDL, which was scaled in the opposite direction (20). Hence, the possible range for SIQALS is between 0 and 8, where a larger value indicates a higher amount of allostatic load. Analysis was restricted to euthyroid subjects (as defined by TSH and FT4 concentrations within their respective laboratory-defined reference ranges) without known thyroid disease, pregnancy or any major comorbidity potentially leading to non-thyroidal illness syndrome. We relied on Jostel’s TSH index (JTI) as a marker for the location of the pituitary-thyroid set point (41). Additionally, estimates for thyroid’s secretory capacity (SPINA-GT) and total deiodinase activity (SPINA-GD) were calculated in order to provide further insights into the physiological roots of potential variations in hormone levels (41).

We tested for associations between allostatic load and markers of thyroid function with unadjusted ordinary least squares (OLS) regression. In these univariate analyses, the multiple components contributing to type 2 allostatic load were expressed as an equally weighted summary score (SIQALS 2) in order to avoid potential issues with collinearity in multivariable analysis. To statistically account for direction of causality and derive more robust estimators in the possible presence of endogenous regressors and feedback (reversed causality), we relied on instrumental variables estimation (IV regression) (42), using the R package AER (43). We selected parameters from the somatic, psychological and social domain as candidates for instrumental variables. Eligible as instruments were parameters that correlated with both SIQALS 2 and JTI, and whose influence could be assumed to be mediated *via* allostatic load only (**Supplementary Tables 3** and **4**). The Durbin-Wu-Hausmann test was used to detect potential endogeneity and to decide if OLS and IV regressions are equally consistent or if IV are to be preferred over OLS models. The Sargan test was performed to test for over-identification, when multiple IVs were used. Additionally, a test for “weak instruments” was performed to decide about the validity of IVs.

## Results

We identified 16 studies investigating TSH and/or thyroid hormone concentrations in subjects with and without PTSD. Two older studies were excluded, since they reported a time interval between trauma and evaluation of thyroid function of a month or less, rendering them incompatible with current definitions of PTSD, and one study was excluded, because only total thyroid hormones but neither TSH nor free thyroid hormones were determined. The remaining 13 studies could be included in the meta-analysis (**Supplementary Figure 1**) (25–27, 44–53).

**Figure 1:**
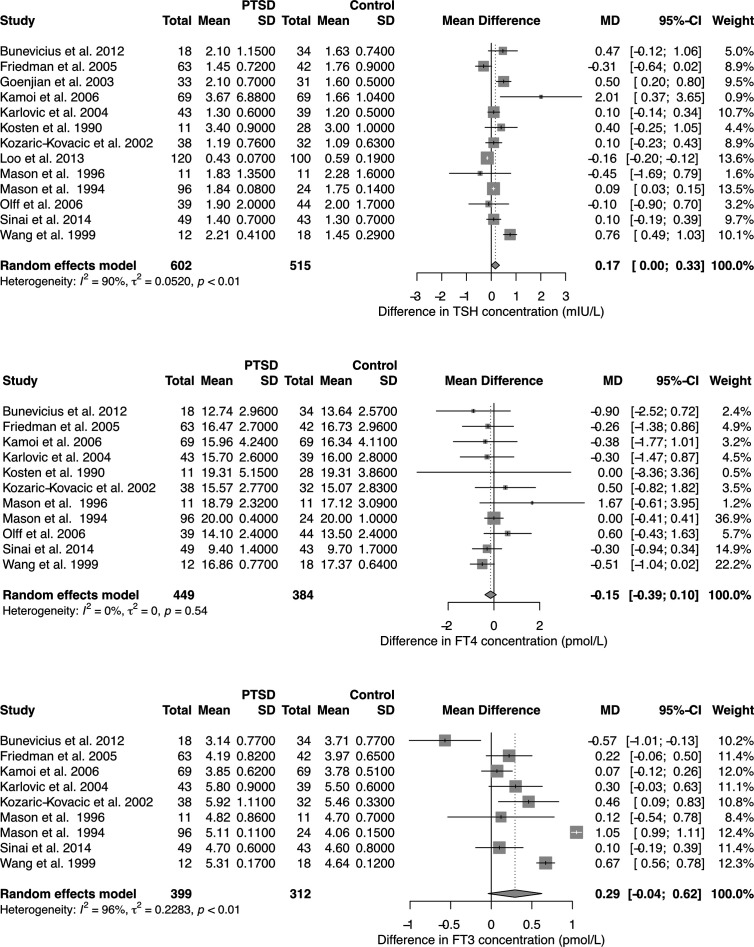
Forest plots showing differences in TSH, FT4, and FT3 concentration between subjects with and without PTSD. See **Supplementary Figures 3–5** for results in chronic PTSD and for total T3 (TT3) and total T4 (TT4) concentration.

With respect to TSH and FT3 concentrations the studies were highly heterogeneous, but the findings were unanimous for FT4 concentrations. Combining the results yields a positive association of PTSD with TSH levels in the random effects model, but unchanged FT4 concentrations (**Figure 1**). FT3 concentration tended to be higher in PTSD.

Meta-regression between concentration differences and time of the exposure to the triggering event revealed a significant correlation for TSH levels (**Supplementary Figure 2**). Accordingly, the observed pooled study effects were even more pronounced in a subset of results with chronic PTSD (24 months or more since the occurrence of the triggering event, **Supplementary Figure 3**). Total T3 concentrations were significantly higher in PTSD compared to controls (**Supplementary Figures 4** and **5**).

Funnel plots (**Supplementary Figure 6**) and tests for small study effects demonstrated that publication bias resulting from less frequent publication of non-significant results does not play a decisive role.

**Figure 2:**
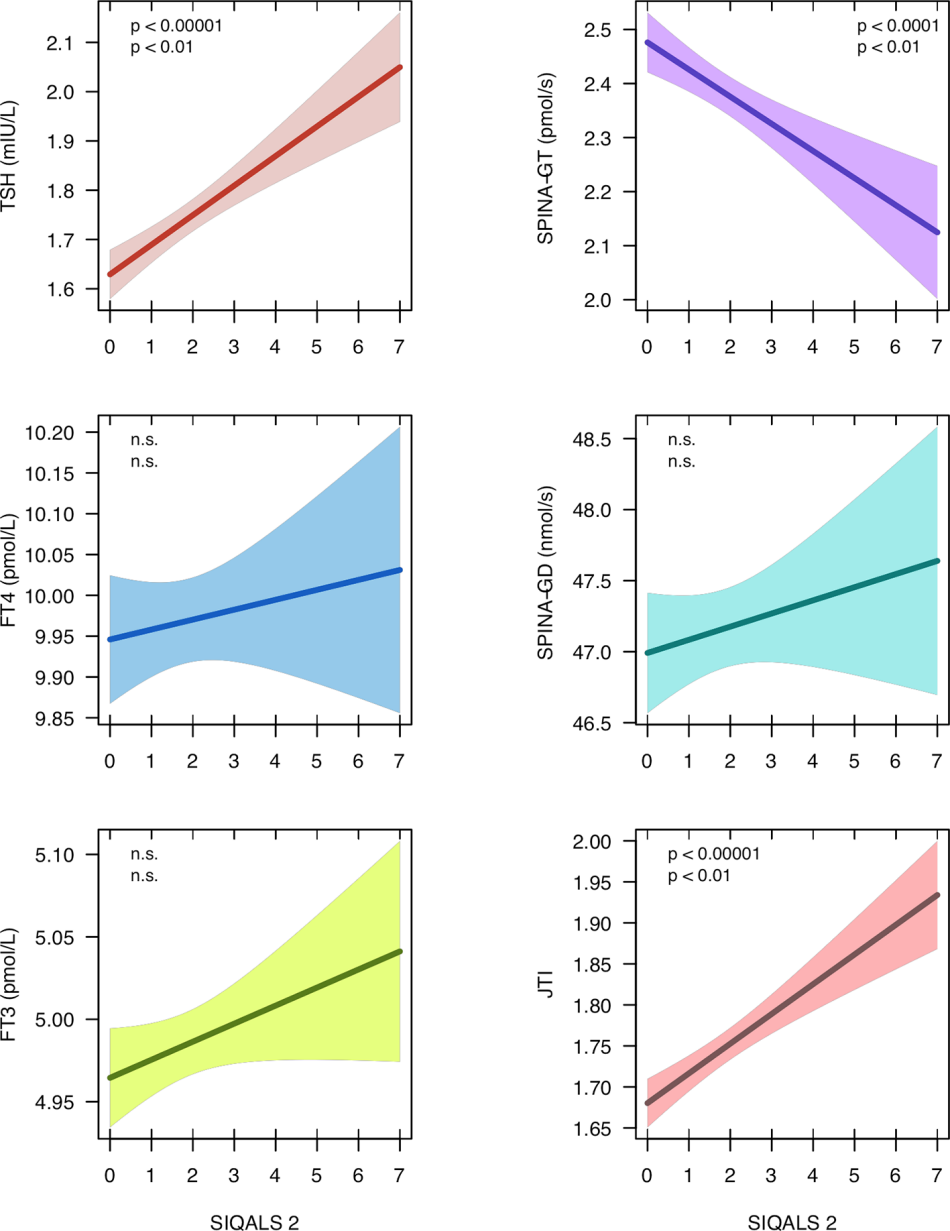
Regression models between type 2 allostatic load (SIQALS 2) and parameters of thyroid homeostasis in 3386 euthyroid subjects. Data were obtained from the National Health and Nutrition Examination Survey (NHANES), 2007 to 2008 (40). Shown is the regression line with 95% confidence bands. In each panel, the first p value refers to unadjusted OLS regression and the second p value to estimates derived from instrumental variable (IV) regression. For regression details, see **Supplementary Tables 2** and **6**.

From the NHANES dataset records of 3386 euthyroid subjects (1579 female, mean ± SD of age 44.0 ± 14.0 years) were eligible. Median and interquartile range (IQR) of SIQALS 2 results were both 2 with a total range between 0 and 7. In the majority of subjects, Jostel’s TSH index was in the reported reference range (1.3 to 4.1) (41, 54) with a mean of 1.75 and SD of 0.57. Allostatic load was significantly positively associated with both TSH concentration and JTI, and negatively with SPINA-GT, as shown in **Figure 2** and **Supplementary Table 2**. JTI weakly, but significantly increased with age, beta coefficient per year ± SE = 3.8e–4 ± 5.9e–5, p < 1e–9. It was also slightly elevated in diabetes and prediabetes (mean ± SEM 1.85 ± 0.03 and 1.88 ± 0.06, resp., vs. 1.74 ± 0.01 and 1.73 ± 0.01, respective p < 0.001 and < 0.02).

Poor socio-economic status (assessed with the family monthly poverty level index) (40, 55), sleep disorder (snoring) and illicit drug usage (cannabis), as reported in the NHANES survey, met above eligibility criteria (**Supplementary Tables 3** and **4**) and were added as instrumental variables. Since in the multivariable model with 3 IVs the Durbin-Wu-Hausmann test did not reveal an advantage of IV over OLS models and the Sargan test suggested over-identification we simplified the model to two predictors (cannabis and snoring, **Supplementary Figure 7**). This model with two IVs confirmed the significant relationship between SIQALS 2 and JTI (beta ± SE = 0.09 ± 0.03, p < 0.01), TSH concentration (beta ± SE = 0.13 ± 0.05, p < 0.01) and SPINA-GT (beta ± SE = –0.16 ± 0.06, p < 0.01). No correlations were observed for FT4 or FT3 concentrations and deiodinase activity (SPINA-GD).

## Discussion

A high-normal or moderately elevated TSH concentration may arise from different causes including setpoint alterations of the homeostatic system in response to PTSD or other causes of allostatic load, as demonstrated here. High SIQALS 2 scores may raise the TSH concentration by 1.04 mIU/L and Jostel’s TSH index by 0.72, compared to low allostatic load. Given the small dispersion of intra-individual variation in TSH concentration this influence is substantial (56). Therefore, the width of the observed broad interindividual reference ranges for TSH and thyroid hormones in population studies may at least partly result from inter-personal differences in allostatic load, age and other cardiovascular risk factors.

In this secondary analysis, we found allostatic load to be unrelated to FT4 or FT3 concentration, but to negatively correlate with maximum thyroid capacity (SPINA-GT). Likewise, PTSD was accompanied by increased TSH concentration, but did not show a clear association to FT4 levels. Interestingly, the association to TSH even grew stronger with a rising time interval since the traumatic event, possibly due to therapeutic interventions addressing a depression-related component of PTSD.

In summary, high-normal or slightly increased TSH concentration, as typically observed in patients with subclinical hypothyroidism, may ensue from two different etiologies, i.e. very early forms of primary hypothyroidism and set point elevation of the otherwise normal feedback control system. At the population level these two mechanisms may be conflated, so that mean concentrations of peripheral thyroid hormones may remain unaltered, although the underlying causal relationships differ.

Our analysis suggests a possible mechanism linking TSH to cardiovascular complications, which is a sensible alternative to the thyroid failure hypothesis by other authors (16). It also well accounts for the U-shaped relationship between TSH concentration and cardiovascular risk, as elsewhere described (14, 15). Hence, a dual etiology may explain the findings of previous studies. To resolve this ambiguity, measurements of peripheral thyroid hormones are required (11) and should be integrated into future studies—not only for the selection of subjects but also for functional assessment (10, 57, 58). Calculated parameters providing biomarkers for the set point of thyroid homeostasis and peripheral hormone metabolism may provide additional insights, especially in the setting of clinical trials (41). This should avoid ambiguities in the interpretation and provide further direction on potential therapeutic intervention (59).

In conclusion, the link between the central stress response and cardiovascular risk is empirically well established (24, 60, 61). Apparently, as demonstrated here, it also involves the central control of thyroid function. Since thyroid hormones have a profound effect on cardiovascular physiology (62–64), the set point of thyroid homeostasis appears to play a more important role than previously assumed.

## Data Availability Statement

The data used for secondary analysis was obtained from the National Health and Nutrition Examination Survey (NHANES) data set, period 2007 to 2008 (https://www.cdc.gov/nchs/nhanes/). The S scripts for generating the figures and meta-analysis, a graphical explanation of instrumental variable regression, supplementary figures and tables with additional results are available as online supplement to this article and from zenodo.org (DOI 10.5281/zenodo.3701232). The protocol for meta-analysis has been registered by PROSPERO with the ID CRD42020208436 and is available from https://www.crd.york.ac.uk/prospero/display_record.php?ID=CRD42020208436.

## Author Contributions

JD drafted a first version of the manuscript. RH, JM, FB, and PM edited the text and contributed additional ideas, material, and text passages. All authors contributed to the article and approved the submitted version.

## Funding

We acknowledge support by the DFG Open Access Publication Funds of the Ruhr-Universität Bochum.

## Conflict of Interest

JD received funding and personal fees by Sanofi-Henning, Hexal AG, Bristol-Myers Squibb, and Pfizer, and is co-owner of the intellectual property rights for the patent “System and Method for Deriving Parameters for Homeostatic Feedback Control of an Individual” (Singapore Institute for Clinical Sciences, Biomedical Sciences Institutes, Application Number 201208940-5, WIPO number WO/2014/088516). The funders had no role in study design, data collection and analysis, decision to publish, or preparation of the manuscript. RH has been paid personal fees in the past for consultancy and code development in general research methods and data analysis.

The remaining authors declare that the research was conducted in the absence of any commercial or financial relationships that could be construed as a potential conflict of interest.
