# Supplementary material for: The Two Faces of Janus: Why Thyrotropin as a Cardiovascular Risk Factor May Be an Ambiguous Target
Source: Front Endocrinol (Lausanne). 2020 Oct 26;11:542710. doi: 10.3389/fendo.2020.542710 (PMC7649136; doi:10.3389/fendo.2020.542710)
Supplement: Supplementary file 1 [file DataSheet_1.pdf]

Supplementary Material

The two faces of Janus: Why thyrotropin as a cardiovascular risk factor may be an ambiguous target.

Johannes W. Dietrich, Rudolf Hoermann, John E. M. Midgley, Friederike Bergen, Patrick Müller*

*** Correspondence:** Corresponding Author: johannes.dietrich@ruhr-uni-bochum.de

Supplementary Code

S Script for basic evaluation of allostatic load and instrumental variable regression. R version 3.5 or newer required (R Project for Statistical Computing, RRID:SCR_001905).

```
# Evaluation of NHANES data with respect to type 2 allostatic load and thyroid function
# Period 2007-2008
# Version 1.0: 20200313 by JWD
# Version 1.1: 20200429 by JWD
# Version 1.2: 20200913 by JWD
# Authors: J. W. Dietrich, R. Hoermann, J. E. M. Midgley, F. Bergen and P. Mueller

# This script uses original data of the NHANES study (period 2007-2008), which are
# available from https://www.cdc.gov/nchs/nhanes/

# The following libraries, some of them coming with R, others being available from
CRAN,
# are necessary:
# MASS, foreign, visreg, SPINA and AER.

# ID: SEQN
# age: RIDAGEYR
# sex: RIAGENDR (1: male, 2: female)
# TSH: LBDTSH1S
# FT4: LBDT4FSI
# FT3: LBDT3FSI
# TT4: LBXTT4
# TT3: LBXTT3
# SPINA-GT: SPINA_GT
# SPINA-GD: SPINA_GD
# TSHI: TSHI
# BMI: BMXBMI
# BSA: BSA
# CorIEx: CORIEX
# Waist Circumference: BMXWAIST
# Urinary Iodine Excretion: URXUIO
# Thyroglobulin antibodies: LBXATG
# Thyroglobulin: LBDTGNSI
# Thyroid Peroxidase antibodies: LBXTPO
# Serum albumin: LBDSALSI
# Serum total protein: LBDSTPSI
# Cholesterol: LBDSCHSI
# Triglycerides: LBDSTRSI
# Total bilirubin: LBDSTBSI
```

```
# Pregnancy status: RIDEXPRG (1 = yes)
# History of cancer: MCQ220
# History of thyroid problem: MCQ160M
# Liver disease: MCQ170L
# Known diabetes: DIQ010

library(MASS);
library(foreign);
if (!require('visreg')) install.packages('visreg'); library('visreg')
if (!require('SPINA')) install.packages('SPINA'); library('SPINA')
if (!require('AER')) install.packages('AER'); library('AER')

output.to.file <- TRUE;

if (Sys.info()[["sysname"]] == "Darwin" | Sys.info()[["machine"]] == "Macintosh")
{ # find the path of this script (macOS only)
  this.frame <- sys.frame(sys.nframe());
  this.dir <- dirname(sys.frame(1)$ofile);
  the.path <- paste(this.dir, "/", sep = "");
} else
  the.path <- getwd();

sem <- function(vector, ...)
{  # standard error of the mean
  stdev <- sd(vector, ...);
  count <- length(na.omit(vector));
  return(stdev/sqrt(count));
}

basic.measures <- function(theFormula, na.rm = TRUE)
{  # delivers a table with basic statistical measures
  if (is.na(charmatch("~", theFormula)))
  {
    theParameter <- theFormula;
    reply <- list(categories = "", overview = "");
    mat1 <- as.matrix(mean(as.numeric(theParameter), na.rm = na.rm));
    mat2 <- as.matrix(sd(as.numeric(theParameter), na.rm = na.rm));
    mat3 <- as.matrix(sem(as.numeric(theParameter), na.rm = na.rm));
    mat4 <- as.matrix(median(as.numeric(theParameter), na.rm = na.rm));
    mat5 <- as.matrix(IQR(as.numeric(theParameter), na.rm = na.rm));
    mat <- cbind(mat1, mat2, mat3, mat4, mat5);
    colnames(mat) <- c("mean", "sd", "sem", "median", "IQR");
    reply$categories <- summary(as.numeric(theParameter), na.rm = na.rm);
    reply$overview <- mat;
    return(reply);
  }
  theParameter <- eval(theFormula[[2]]);  # extract parts from formula notation
  theCriterion <- eval(theFormula[[3]]);
  reply <- list(categories = "", overview = "");
  mat1 <- as.matrix(tapply(as.numeric(theParameter), (as.factor(theCriterion)), mean,
na.rm = na.rm));
  mat2 <- as.matrix(tapply(as.numeric(theParameter), (as.factor(theCriterion)), sd,
na.rm = na.rm));
  mat3 <- as.matrix(tapply(as.numeric(theParameter), (as.factor(theCriterion)), sem,
na.rm = na.rm));
  mat4 <- as.matrix(tapply(as.numeric(theParameter), (as.factor(theCriterion)), median,
na.rm = na.rm));
  mat5 <- as.matrix(tapply(as.numeric(theParameter), (as.factor(theCriterion)), IQR,
na.rm = na.rm));
  mat <- cbind(mat1, mat2, mat3, mat4, mat5);
  colnames(mat) <- c("mean", "sd", "sem", "median", "IQR");
  reply$categories <- tapply(as.numeric(theParameter), (as.factor(theCriterion)),
summary);
  reply$overview <- mat;
  return(reply);
}

annotated.t.test <- function(theFormula)
{ # extended version of t-test that also delivers basic measures
```

```
  reply <- list(formula = theFormula, test.results = "", description = list());
  reply$test.results <- t.test(theFormula);
  reply$description <- basic.measures(theFormula);
  return(reply);
}

sig.sym <- function(p.value, sig.marks = "symbols", draw.always = FALSE, digits = 4)
{ # delivers symbols or rounded numbers for significance level.
  if ((p.value < 0.05) | draw.always)
  {
    if (sig.marks == "symbols")
      sig.label <- (if (p.value < 0.00001) expression("*****") else if (p.value <
0.0001) expression("****") else if (p.value < 0.001) expression("***") else if (p.value
< 0.01) expression("**") else if (p.value < 0.05) expression("*") else
expression("n.s."))
    else if (sig.marks == "text")
      sig.label <- (if (p.value < 0.00001) expression("p < 0.00001") else if (p.value <
0.0001) expression("p < 0.0001") else if (p.value < 0.001) expression("p < 0.001") else
if (p.value < 0.01) expression("p < 0.01") else if (p.value < 0.05) expression("p <
0.05") else expression("n.s."))
    else if (sig.marks == "exact")
    sig.label <- paste("p =", round(p.value, digits = digits));
      return(as.character(sig.label));
  }
}

annotated.barplot <- function(heights, categories, xlab = "", ylab = "", names.arg =
NA, main = "", sub = "", ylim = NULL, meanbar = FALSE, bar.col = NULL, whisk.col =
par("fg"), new = FALSE, ...)
{ # extended version of barplot containing whiskers for dispersion
  the.heights <- tapply(heights, categories, mean, na.rm = TRUE);
  the.whiskers <- tapply(heights, categories, sem, na.rm = TRUE);
  if (is.null(ylim))
  {
    if (min(the.heights, na.rm = TRUE) < 0)
      ylim = c(min(the.heights, na.rm = TRUE), max(the.heights, na.rm = TRUE) + 7 *
max(the.whiskers, na.rm = TRUE)) else
      ylim = c(0, max(the.heights, na.rm = TRUE) + 7 * max(the.whiskers, na.rm = TRUE,
na.rm = TRUE));
  }
  the.plot <- barplot(the.heights, xlab = xlab, ylab = ylab, names.arg = names.arg,
ylim = ylim, main = main, sub = sub, new = new, ...);
  arrows(the.plot, the.heights + the.whiskers, the.plot, the.heights, angle = 90, code
= 1, length = 0.05, col = whisk.col);
  arrows(the.plot, the.heights - the.whiskers, the.plot, the.heights, angle = 90, code
= 1, length = 0.05, col = whisk.col);
  if (meanbar) rect(the.plot - 0.3, the.heights - (ylim[2] - ylim[1]) / 100, the.plot +
0.3, the.heights + (ylim[2] - ylim[1]) / 100, col = bar.col);
  return(list(barplot = the.plot, heights = the.heights, whiskers = the.whiskers));
}

file.thyroid <- paste(the.path, "THYROD_E.XPT", sep = ""); # thyroid parameters
file.iodine <- paste(the.path, "UIO_E.XPT", sep = ""); # urinary iodine
file.demographics <- paste(the.path, "DEMO_E.XPT", sep = ""); # demographics
file.bp <- paste(the.path, "BPX_E.XPT", sep = ""); # blood pressure
file.tchol <- paste(the.path, "TCHOL_E.XPT", sep = ""); # total cholesterol
file.hdl <- paste(the.path, "HDL_E.XPT", sep = ""); # HDL
file.hba1c <- paste(the.path, "GHB_E.XPT", sep = ""); # HbA1c
file.crp <- paste(the.path, "CRP_E.XPT", sep = ""); # CRP
file.bodym <- paste(the.path, "BMX_E.XPT", sep = ""); # body measures
file.conditions <- paste(the.path, "MCQ_E.XPT", sep = ""); # medical conditions
file.gluc.insu <- paste(the.path, "GLU_E.XPT", sep = ""); # Fasting glucose and insulin
file.biochemistry <- paste(the.path, "BIOPRO_E.XPT", sep = ""); # Standard biochemistry
file.diabetes <- paste(the.path, "DIQ_E.XPT", sep = ""); # Diabetes
file.drugs <- paste(the.path, "DUQ_E.XPT", sep = ""); # Drug use
file.sleep.quality <- paste(the.path, "SLQ_E.XPT", sep = ""); # Sleep disorders
file.health.status <- paste(the.path, "HSQ_E.XPT", sep = ""); # Current health status
file.income <- paste(the.path, "INQ_E.XPT", sep = ""); # income

plot.file <- paste(the.path, "plots.pdf", sep = "");
```

```
log.file <- paste(the.path, "log.txt", sep = "");
export.file <- paste(the.path, "NHANES_2007.csv", sep = "");

mat.thyroid <- read.xport(file.thyroid);
mat.iodine <- read.xport(file.iodine);
mat.demo_e <- read.xport(file.demographics);
mat.bp <- read.xport(file.bp);
mat.thcol <- read.xport(file.tchol);
mat.hdl <- read.xport(file.hdl);
mat.hba1c <- read.xport(file.hba1c);
mat.crp <- read.xport(file.crp);
mat.bodym <- read.xport(file.bodym);
mat.conditions <- read.xport(file.conditions);
mat.gluc.insu <- read.xport(file.gluc.insu);
mat.biochemistry <- read.xport(file.biochemistry);
mat.diabetes <- read.xport(file.diabetes);
mat.drugs <- read.xport(file.drugs);
mat.sleep.quality <- read.xport(file.sleep.quality);
mat.health.status <- read.xport(file.health.status);
mat.income <- read.xport(file.income);

# The following lines "curate" data frames, so that for selected variables missing
# or invalid values are replaced by NA:

mat.health.status$mentally.impaired.days <- mat.health.status$HSQ480;
mat.health.status$mentally.impaired.days[which(mat.health.status$HSQ480 > 30)] <- NA;
mat.health.status$days.of.anxiety <- mat.health.status$HSQ496;
mat.health.status$days.of.anxiety[which(mat.health.status$HSQ496 > 30)] <- NA;

mat.sleep.quality$SAS <- 0;
mat.sleep.quality$SAS[which(mat.sleep.quality$SLQ070A == 1)] <- 1;
mat.sleep.quality$SLD.c <- mat.sleep.quality$SLD010H;
mat.sleep.quality$SLD.c[which(mat.sleep.quality$SLD010H > 12)] <- NA;
mat.sleep.quality$snoring.c <- mat.sleep.quality$SLQ030;
mat.sleep.quality$snoring.c[which(mat.sleep.quality$SLQ030 > 3)] <- NA;

mat.drugs$DUQ220Q.c <- mat.drugs$DUQ220Q; # marijuana or hashish
mat.drugs$DUQ220Q.c[which(mat.drugs$DUQ220Q > 365)] <- NA;
mat.drugs$DUQ220Q.c[which(mat.drugs$DUQ220U == 2)] <-
mat.drugs$DUQ220Q.c[which(mat.drugs$DUQ220U == 2)] * 7;
mat.drugs$DUQ220Q.c[which(mat.drugs$DUQ220U == 3)] <-
mat.drugs$DUQ220Q.c[which(mat.drugs$DUQ220U == 3)] * 30;
mat.drugs$DUQ220Q.c[which(mat.drugs$DUQ220U == 4)] <-
mat.drugs$DUQ220Q.c[which(mat.drugs$DUQ220U == 4)] * 365;
mat.drugs$DUQ220Q.c[which(mat.drugs$DUQ220U > 4)] <- NA;

mat.drugs$DUQ200.c <- mat.drugs$DUQ200;
mat.drugs$DUQ200.c[which(mat.drugs$DUQ200 > 2)] <- NA;

mat.drugs$DUQ270Q.c <- mat.drugs$DUQ270Q; # cocaine
mat.drugs$DUQ270Q.c[which(mat.drugs$DUQ270Q > 365)] <- NA;
mat.drugs$DUQ270Q.c[which(mat.drugs$DUQ270U == 2)] <-
mat.drugs$DUQ270Q.c[which(mat.drugs$DUQ270U == 2)] * 7;
mat.drugs$DUQ270Q.c[which(mat.drugs$DUQ270U == 3)] <-
mat.drugs$DUQ270Q.c[which(mat.drugs$DUQ270U == 3)] * 30;
mat.drugs$DUQ270Q.c[which(mat.drugs$DUQ270U == 4)] <-
mat.drugs$DUQ270Q.c[which(mat.drugs$DUQ270U == 4)] * 365;
mat.drugs$DUQ270Q.c[which(mat.drugs$DUQ270U > 4)] <- NA;

mat.drugs$DUQ272.c <- mat.drugs$DUQ272; # cocaine
mat.drugs$DUQ272.c[which(mat.drugs$DUQ272 > 6)] <- 0;

mat.drugs$DUQ280.c <- mat.drugs$DUQ280; # cocaine
mat.drugs$DUQ280.c[which(is.na(mat.drugs$DUQ280))] <- 0;
mat.drugs$DUQ280.c[which(mat.drugs$DUQ280 > 30)] <- 0;

mat.drugs$DUQ310Q.c <- mat.drugs$DUQ310Q; # heroin
mat.drugs$DUQ310Q.c[which(mat.drugs$DUQ310Q > 365)] <- NA;
mat.drugs$DUQ310Q.c[which(mat.drugs$DUQ310U == 2)] <-
mat.drugs$DUQ310Q.c[which(mat.drugs$DUQ310U == 2)] * 7;
```

```
mat.drugs$DUQ310Q.c[which(mat.drugs$DUQ310U == 3)] <-
mat.drugs$DUQ310Q.c[which(mat.drugs$DUQ310U == 3)] * 30;
mat.drugs$DUQ310Q.c[which(mat.drugs$DUQ310U == 4)] <-
mat.drugs$DUQ310Q.c[which(mat.drugs$DUQ310U == 4)] * 365;
mat.drugs$DUQ310Q.c[which(mat.drugs$DUQ310U > 4)] <- NA;

mat.drugs$DUQ320.c <- mat.drugs$DUQ320; # heroin
mat.drugs$DUQ320.c[which(is.na(mat.drugs$DUQ320))] <- 0;
mat.drugs$DUQ320.c[which(mat.drugs$DUQ320 > 30)] <- 0;

mat.drugs$DUQ350Q.c <- mat.drugs$DUQ350Q; # methamphetamine
mat.drugs$DUQ350Q.c[which(mat.drugs$DUQ350Q > 365)] <- NA;
mat.drugs$DUQ350Q.c[which(mat.drugs$DUQ350U == 2)] <-
mat.drugs$DUQ350Q.c[which(mat.drugs$DUQ350U == 2)] * 7;
mat.drugs$DUQ350Q.c[which(mat.drugs$DUQ350U == 3)] <-
mat.drugs$DUQ350Q.c[which(mat.drugs$DUQ350U == 3)] * 30;
mat.drugs$DUQ350Q.c[which(mat.drugs$DUQ350U == 4)] <-
mat.drugs$DUQ350Q.c[which(mat.drugs$DUQ350U == 4)] * 365;
mat.drugs$DUQ350Q.c[which(mat.drugs$DUQ350U > 4)] <- NA;

mat.drugs$DUQ352.c <- mat.drugs$DUQ352; # methamphetamine
mat.drugs$DUQ352.c[which(mat.drugs$DUQ352 > 6)] <- 0;

mat.drugs$DUQ360.c <- mat.drugs$DUQ360; # methamphetamine
mat.drugs$DUQ360.c[which(is.na(mat.drugs$DUQ360))] <- 0;
mat.drugs$DUQ360.c[which(mat.drugs$DUQ360 > 30)] <- 0;

mat.iodine$CORIEX <- mat.iodine$URXUIO / (mat.iodine$URXUCR * 10 / 1000); # iodine in
mcg/L, creatinine in mg/dl
mat.bodym$BSA <- 0.007184 * mat.bodym$BMXHT^0.728 * mat.bodym$BMXWT^0.425;

mat.demo_e$RIDEXPRG.2 <- mat.demo_e$RIDEXPRG; # Pregnancy
mat.demo_e$RIDEXPRG.2[is.na(mat.demo_e$RIDEXPRG)] <- 0;

mat.demo_e$DMDEDUC2.c <- mat.demo_e$DMDEDUC2; # Education Level (adults 20+)
mat.demo_e$DMDEDUC2.c[which(mat.demo_e$DMDEDUC2 > 5)] <- NA; # Exclude invalid data

mat.demo_e$DMDHREDU.c <- mat.demo_e$DMDHREDU; # Ref. person Education Level (adults
20+)
mat.demo_e$DMDHREDU.c[which(mat.demo_e$DMDHREDU > 5)] <- NA; # Exclude invalid data

mat.demo_e$DMQMILIT.c <- mat.demo_e$DMQMILIT; # Served in US armed forces
mat.demo_e$DMQMILIT.c[which(mat.demo_e$DMDEDUC2 > 5)] <- NA; # Exclude invalid data

mat.demo_e$INDHHIN2.c <- mat.demo_e$INDHHIN2; # Annual household income
mat.demo_e$INDHHIN2.c[which(mat.demo_e$INDHHIN2 > 15)] <- NA; # Exclude invalid data
mat.demo_e$INDHHIN2.c[which(mat.demo_e$INDHHIN2 == 12)] <- NA; # Exclude invalid data
mat.demo_e$INDHHIN2.c[which(mat.demo_e$INDHHIN2 == 13)] <- NA; # Exclude invalid data

mat.demo_e$INDFMIN2.c <- mat.demo_e$INDFMIN2; # Annual family income
mat.demo_e$INDFMIN2.c[which(mat.demo_e$INDFMIN2 > 15)] <- NA; # Exclude invalid data
mat.demo_e$INDFMIN2.c[which(mat.demo_e$INDFMIN2 == 12)] <- NA; # Exclude invalid data
mat.demo_e$INDFMIN2.c[which(mat.demo_e$INDFMIN2 == 13)] <- NA; # Exclude invalid data

mat.diabetes$DIQ010.c <- mat.diabetes$DIQ010; # Diabetes
mat.diabetes$DIQ010.c[which(mat.diabetes$DIQ010 > 2)] <- NA;
mat.diabetes$DIQ010.c <- 2 - mat.diabetes$DIQ010.c;

mat.diabetes$DIQ160.c <- mat.diabetes$DIQ160; # Prediabetes
mat.diabetes$DIQ160.c[which(mat.diabetes$DIQ160 > 2)] <- NA;
mat.diabetes$DIQ160.c <- 2 - mat.diabetes$DIQ160.c;

mat.thyroid$LBDT3FSI <- mat.thyroid$LBXT3F * 1.54;
mat.thyroid$SPINA_GT <- SPINA.GT(mat.thyroid$LBDTSH1S, mat.thyroid$LBDT4FSI);
mat.thyroid$SPINA_GD <- SPINA.GD(mat.thyroid$LBDT4FSI, mat.thyroid$LBDT3FSI);
mat.thyroid$TSHI <- estimated.TSHI(mat.thyroid$LBDTSH1S, mat.thyroid$LBDT4FSI);
mat.thyroid$sTSHI <- estimated.sTSHI(mat.thyroid$LBDTSH1S, mat.thyroid$LBDT4FSI);

# Deliver final matrices by merging the partial data frames from NHANES
```

```
mat.allo.1 <- merge(mat.bp, mat.thcol, by = "SEQN");
mat.allo.2 <- merge(mat.allo.1, mat.hdl, by = "SEQN");
mat.allo.3 <- merge(mat.allo.2, mat.hba1c, by = "SEQN");
mat.allo.4 <- merge(mat.allo.3, mat.crp, by = "SEQN");
mat.allo.5 <- merge(mat.allo.4, mat.drugs, by = "SEQN");
mat.allo.6 <- merge(mat.allo.5, mat.sleep.quality, by = "SEQN");
mat.allo.7 <- merge(mat.allo.6, mat.health.status, by = "SEQN");
mat.allo.8 <- merge(mat.allo.7, mat.income, by = "SEQN");

mat.thyroid.1 <- merge(mat.iodine, mat.thyroid, by = "SEQN");
mat.thyroid.2 <- merge(mat.conditions, mat.thyroid.1, by = "SEQN");
mat.thyroid.3 <- merge(mat.bodym, mat.thyroid.2, by = "SEQN");
mat.thyroid.4 <- merge(mat.demo_e, mat.thyroid.3, by = "SEQN");
mat.thyroid.5 <- merge(mat.biochemistry, mat.thyroid.4, by = "SEQN");
mat.thyroid.6 <- merge(mat.diabetes, mat.thyroid.5, by = "SEQN");

# Select normal cases, not affected by thyroid disease or conditions leading to
# type 1 allostatic load:

mat.thyroid.normal <- mat.thyroid.6[which((mat.thyroid.6$MCQ160M == 2) &
(mat.thyroid.6$RIDEXPRG.2 != 1)),]; # no thyroid disease
mat.thyroid.normal.noNTIS <- mat.thyroid.normal[which((mat.thyroid.normal$MCQ220 !=1) &
((mat.thyroid.normal$RIDEXPRG !=1) | is.na(mat.thyroid.normal$RIDEXPRG)) &
((mat.thyroid.normal$MCQ170L !=1) | is.na(mat.thyroid.normal$MCQ170L))),]; # no
pregnancy, cancer or liver condition

mat.thyroid.normal.allo <- merge(mat.thyroid.normal.noNTIS, mat.allo.8, by = "SEQN");

# Prepare calculation of SIQALS 2 score:

mat.thyroid.normal.allo$PR.quartiles <- cut(mat.thyroid.normal.allo$BPXPLS,
quantile(mat.thyroid.normal.allo$BPXPLS, c(0, 0.25, 0.5, 0.75, 1), na.rm = TRUE),
include.lowest = TRUE);
mat.thyroid.normal.allo$PR.score <- 0;
mat.thyroid.normal.allo$PR.score[which(mat.thyroid.normal.allo$PR.quartiles ==
levels(mat.thyroid.normal.allo$PR.quartiles)[4])] <- 1;

mat.thyroid.normal.allo$BPXSYM <- rowMeans(cbind(mat.thyroid.normal.allo$BPXSY1,
mat.thyroid.normal.allo$BPXSY2, mat.thyroid.normal.allo$BPXSY3,
mat.thyroid.normal.allo$BPXSY4), na.rm = TRUE);
mat.thyroid.normal.allo$SBP.quartiles <- cut(mat.thyroid.normal.allo$BPXSYM,
quantile(mat.thyroid.normal.allo$BPXSYM, c(0, 0.25, 0.5, 0.75, 1), na.rm = TRUE),
include.lowest = TRUE);
mat.thyroid.normal.allo$SBP.score <- 0;
mat.thyroid.normal.allo$SBP.score[which(mat.thyroid.normal.allo$SBP.quartiles ==
levels(mat.thyroid.normal.allo$SBP.quartiles)[4])] <- 1;

mat.thyroid.normal.allo$BPXDIM <- rowMeans(cbind(mat.thyroid.normal.allo$BPXDI1,
mat.thyroid.normal.allo$BPXDI2, mat.thyroid.normal.allo$BPXDI3,
mat.thyroid.normal.allo$BPXDI4), na.rm = TRUE);
mat.thyroid.normal.allo$DBP.quartiles <- cut(mat.thyroid.normal.allo$BPXDIM,
quantile(mat.thyroid.normal.allo$BPXDIM, c(0, 0.25, 0.5, 0.75, 1), na.rm = TRUE),
include.lowest = TRUE);
mat.thyroid.normal.allo$DBP.score <- 0;
mat.thyroid.normal.allo$DBP.score[which(mat.thyroid.normal.allo$DBP.quartiles ==
levels(mat.thyroid.normal.allo$DBP.quartiles)[4])] <- 1;

mat.thyroid.normal.allo$TC.quartiles <- cut(mat.thyroid.normal.allo$LBDTCSI,
quantile(mat.thyroid.normal.allo$LBDTCSI, c(0, 0.25, 0.5, 0.75, 1), na.rm = TRUE),
include.lowest = TRUE);
mat.thyroid.normal.allo$TC.score <- 0;
mat.thyroid.normal.allo$TC.score[which(mat.thyroid.normal.allo$TC.quartiles ==
levels(mat.thyroid.normal.allo$TC.quartiles)[4])] <- 1;

mat.thyroid.normal.allo$HDL.quartiles <- cut(mat.thyroid.normal.allo$LBDHDDSI,
quantile(mat.thyroid.normal.allo$LBDHDDSI, c(0, 0.25, 0.5, 0.75, 1), na.rm = TRUE),
include.lowest = TRUE);
mat.thyroid.normal.allo$HDL.score <- 0;
mat.thyroid.normal.allo$HDL.score[which(mat.thyroid.normal.allo$HDL.quartiles ==
levels(mat.thyroid.normal.allo$HDL.quartiles)[1])] <- 1;
```

```
mat.thyroid.normal.allo$BMI.quartiles <- cut(mat.thyroid.normal.allo$BMXBMI,
quantile(mat.thyroid.normal.allo$BMXBMI, c(0, 0.25, 0.5, 0.75, 1), na.rm = TRUE),
include.lowest = TRUE);
mat.thyroid.normal.allo$BMI.score <- 0;
mat.thyroid.normal.allo$BMI.score[which(mat.thyroid.normal.allo$BMI.quartiles ==
levels(mat.thyroid.normal.allo$BMI.quartiles)[4])] <- 1;

mat.thyroid.normal.allo$HbA1c.quartiles <- cut(mat.thyroid.normal.allo$LBXGH,
quantile(mat.thyroid.normal.allo$LBXGH, c(0, 0.25, 0.5, 0.75, 1), na.rm = TRUE),
include.lowest = TRUE);
mat.thyroid.normal.allo$HbA1c.score <- 0;
mat.thyroid.normal.allo$HbA1c.score[which(mat.thyroid.normal.allo$HbA1c.quartiles ==
levels(mat.thyroid.normal.allo$HbA1c.quartiles)[4])] <- 1;

mat.thyroid.normal.allo$CRP.quartiles <- cut(mat.thyroid.normal.allo$LBXCRP,
quantile(mat.thyroid.normal.allo$LBXCRP, c(0, 0.25, 0.5, 0.75, 1), na.rm = TRUE),
include.lowest = TRUE);
mat.thyroid.normal.allo$CRP.score <- 0;
mat.thyroid.normal.allo$CRP.score[which(mat.thyroid.normal.allo$CRP.quartiles ==
levels(mat.thyroid.normal.allo$CRP.quartiles)[4])] <- 1;

# Calculate SIQALS 2 score:

mat.thyroid.normal.allo$SIQALS.2 <- mat.thyroid.normal.allo$PR.score +
mat.thyroid.normal.allo$SBP.score + mat.thyroid.normal.allo$DBP.score +
mat.thyroid.normal.allo$TC.score + mat.thyroid.normal.allo$HDL.score +
mat.thyroid.normal.allo$BMI.score + mat.thyroid.normal.allo$HbA1c.score +
mat.thyroid.normal.allo$CRP.score;

# Select euthyroid subjects (according to NHANES-quoted reference ranges):

mat.thyroid.eu.allo <- mat.thyroid.normal.allo[which((mat.thyroid.normal.allo$LBXTSH1
>= 0.34) & (mat.thyroid.normal.allo$LBXTSH1 <= 5.6) & (mat.thyroid.normal.allo$LBXT4F
>= 0.6) & (mat.thyroid.normal.allo$LBXT4F <= 1.6)),];

# Evaluation:

if (output.to.file)
{
  pdf(plot.file);
  sink(log.file, split = TRUE);
}

cat("\nType 2 allostatic load and thyroid function:");
cat("\n--------------------------------------------\n");

attach(mat.thyroid.eu.allo);

cat("\nBasic statistics:");
cat("\n-----------------\n");

cat("\nSex:\n");
print(table(RIAGENDR));

cat("\nAge:\n");
print(basic.measures(RIDAGEEX / 12));

cat("\nSIQALS 2:\n");
print(basic.measures(SIQALS.2));

cat("\nJTI (TSHI):\n");
print(basic.measures(TSHI));

cat("\nOLS statistics:");
cat("\n---------------\n");

model.1 <- lm(LBDTSH1S ~ SIQALS.2);
print(summary(model.1));
p.model.1 <- summary(model.1)$coefficients[2, 4];
```

```
model.2 <- lm(SPINA_GT ~ SIQALS.2);
print(summary(model.2));
p.model.2 <- summary(model.2)$coefficients[2, 4];

model.3 <- lm(LBDT4FSI ~ SIQALS.2);
print(summary(model.3));
p.model.3 <- summary(model.3)$coefficients[2, 4];

model.4 <- lm(SPINA_GD ~ SIQALS.2);
print(summary(model.4));
p.model.4 <- summary(model.4)$coefficients[2, 4];

model.5 <- lm(LBDT3FSI ~ SIQALS.2);
print(summary(model.5));
p.model.5 <- summary(model.5)$coefficients[2, 4];

model.6 <- lm(TSHI ~ SIQALS.2);
print(summary(model.6));
p.model.6 <- summary(model.6)$coefficients[2, 4];

cat("\nEver told you have diabetes?\n\n");
print(annotated.t.test(TSHI ~ DIQ010.c));
print(annotated.t.test(SPINA_GD ~ DIQ010.c));
cat("\nEver told you have prediabetes?\n\n");
print(annotated.t.test(TSHI ~ DIQ160.c));
print(annotated.t.test(SPINA_GD ~ DIQ160.c));

cat("\nCorrelation to age:\n\n");
print(summary(lm(TSHI ~ RIDAGEEX)));

cat("\nIV statistics:");
cat("\n--------------\n");

cat("\nPotential instrumental variables:");
cat("\n---------------------------------\n");

cat("\nSensible candidates should correlate to both SIQALS 2 and TSHI, but it should
be");
cat("\nreasonably assumed that the correlation to TSHI is mediated via allostasis
only.");

cat("\n\nEducation (adults 20+):\n");
print(summary(lm(SIQALS.2 ~ DMDEDUC2.c)));
print(summary(lm(TSHI ~ DMDEDUC2.c)));

cat("\nFamily monthly poverty level index:\n");
print(summary(lm(SIQALS.2 ~ INDFMMPI)));
print(summary(lm(TSHI ~ INDFMMPI)));

cat("\nSnoring:\n");
print(summary(lm(SIQALS.2 ~ snoring.c)));
print(summary(lm(TSHI ~ snoring.c)));

cat("\nDays of anxiety:\n");
print(summary(lm(SIQALS.2 ~ days.of.anxiety)));
print(summary(lm(TSHI ~ days.of.anxiety)));

cat("\nLast day of cannabis (marijuana or hashish) use:\n");
print(summary(lm(SIQALS.2 ~ DUQ220Q.c)));
print(summary(lm(TSHI ~ DUQ220Q.c)));

cat("\nDo the candidate instrumental variables correlate to FT4?\n");
cat("If not correlated they aren't associated to the error term.\n");

print(summary(lm(LBDT4FSI ~ INDFMMPI)));
print(summary(lm(LBDT4FSI ~ snoring.c)));
print(summary(lm(LBDT4FSI ~ DUQ220Q.c)));

cat("\nIV regression:");
```

```
cat("\n--------------\n");

cat("\nInstrumental variable: Family monthly poverty level index:\n");
print(summary(iv.model.JTI.u1 <- ivreg(TSHI ~ SIQALS.2 | INDFMMPI), diagnostics =
TRUE));
slope.JTI.cannabis <- iv.model.JTI.u1$coefficients[[2]];

cat("\nInstrumental variable: Last day of cannabis (marijuana or hashish) use:\n");
print(summary(iv.model.JTI.u2 <- ivreg(TSHI ~ SIQALS.2 | DUQ220Q.c), diagnostics =
TRUE));
slope.JTI.cannabis <- iv.model.JTI.u2$coefficients[[2]];

cat("\nInstrumental variable: Snoring:\n");
print(summary(iv.model.JTI.u3 <- ivreg(TSHI ~ SIQALS.2 | snoring.c), diagnostics =
TRUE));
slope.JTI.snoring <- iv.model.JTI.u3$coefficients[[2]];

cat("\nMultiple instrumental variables:\n");
print(summary(iv.model.JTI.m21 <- ivreg(TSHI ~ SIQALS.2 | DUQ220Q.c + snoring.c),
diagnostics = TRUE));
slope.JTI.multi.21 <- iv.model.JTI.m21$coefficients[[2]];
p.JTI.multi.21 <- summary(iv.model.JTI.m21)$coefficients[2,4];

print(summary(iv.model.JTI.m22 <- ivreg(TSHI ~ SIQALS.2 | INDFMMPI + snoring.c),
diagnostics = TRUE));
slope.JTI.multi.22 <- iv.model.JTI.m22$coefficients[[2]];

print(summary(iv.model.JTI.m23 <- ivreg(TSHI ~ SIQALS.2 | DUQ220Q.c + INDFMMPI),
diagnostics = TRUE));
slope.JTI.multi.23 <- iv.model.JTI.m23$coefficients[[2]];

print(summary(iv.model.TSH.m21 <- ivreg(LBXTSH1 ~ SIQALS.2 | DUQ220Q.c + snoring.c),
diagnostics = TRUE));
slope.TSH.multi.21 <- iv.model.TSH.m21$coefficients[[2]];
p.TSH.multi.21 <- summary(iv.model.TSH.m21)$coefficients[2,4];

print(summary(iv.model.FT4.m21 <- ivreg(LBDT4FSI ~ SIQALS.2 | DUQ220Q.c + snoring.c),
diagnostics = TRUE));
slope.FT4.multi.21 <- iv.model.FT4.m21$coefficients[[2]];
p.FT4.multi.21 <- summary(iv.model.FT4.m21)$coefficients[2,4];

print(summary(iv.model.FT3.m21 <- ivreg(LBDT3FSI ~ SIQALS.2 | DUQ220Q.c + snoring.c),
diagnostics = TRUE));
slope.FT3.multi.21 <- iv.model.FT3.m21$coefficients[[2]];
p.FT3.multi.21 <- summary(iv.model.FT3.m21)$coefficients[2,4];

print(summary(iv.model.GT.m21 <- ivreg(SPINA_GT ~ SIQALS.2 | DUQ220Q.c + snoring.c),
diagnostics = TRUE));
slope.GT.multi.21 <- iv.model.GT.m21$coefficients[[2]];
p.GT.multi.21 <- summary(iv.model.GT.m21)$coefficients[2,4];

print(summary(iv.model.GD.m21 <- ivreg(SPINA_GD ~ SIQALS.2 | DUQ220Q.c + snoring.c),
diagnostics = TRUE));
slope.GD.multi.21 <- iv.model.GD.m21$coefficients[[2]];
p.GD.multi.21 <- summary(iv.model.GD.m21)$coefficients[2,4];

print(summary(iv.model.JTI.m3 <- ivreg(TSHI ~ SIQALS.2 | DUQ220Q.c + snoring.c +
INDFMMPI), diagnostics = TRUE));
slope.JTI.multi.3 <- iv.model.JTI.m3$coefficients[[2]];
p.JTI.multi.3 <- summary(iv.model.JTI.m3)$coefficients[2,4];

print(summary(iv.model.TSH.m3 <- ivreg(LBXTSH1 ~ SIQALS.2 | DUQ220Q.c + snoring.c +
INDFMMPI), diagnostics = TRUE));
p.TSH.multi.3 <- summary(iv.model.TSH.m3)$coefficients[2,4];

print(summary(iv.model.FT4.m3 <- ivreg(LBDT4FSI ~ SIQALS.2 | DUQ220Q.c + snoring.c +
INDFMMPI), diagnostics = TRUE));
p.FT4.multi.3 <- summary(iv.model.FT4.m3)$coefficients[2,4];
```

```
print(summary(iv.model.FT3.m3 <- ivreg(LBDT3FSI ~ SIQALS.2 | DUQ220Q.c + snoring.c +
INDFMMPI), diagnostics = TRUE));
p.FT3.multi.3 <- summary(iv.model.FT3.m3)$coefficients[2,4];

print(summary(iv.model.GT.m3 <- ivreg(SPINA_GT ~ SIQALS.2 | DUQ220Q.c + snoring.c +
INDFMMPI), diagnostics = TRUE));
p.GT.multi.3 <- summary(iv.model.GT.m3)$coefficients[2,4];

print(summary(iv.model.GD.m3 <- ivreg(SPINA_GD ~ SIQALS.2 | DUQ220Q.c + snoring.c +
INDFMMPI), diagnostics = TRUE));
p.GD.multi.3 <- summary(iv.model.GD.m3)$coefficients[2,4];

print(paste("n =", nrow(mat.thyroid.eu.allo)));

oldpar <- par(mfrow = c(3, 2), mar = c(4, 4, 0, 5) + 0.2, oma = c(1, 5, 0, 3));

visreg(model.1, xlab = "", ylab = "", partial = FALSE, fill = list(col = "mistyrose2"),
line = list(col = "tomato3"));
legend.1 <- sig.sym(p.model.1, sig.marks = "text", draw.always = TRUE);
legend.2 <- sig.sym(p.TSH.multi.21 , sig.marks = "text", draw.always = TRUE);
legend("topleft", legend = paste(legend.1, "\n", legend.2, sep = ""), bty = "n", cex =
0.9);
title(ylab="TSH (mIU/L)", line=3.3, cex.lab=1.0)

visreg(model.2, xlab = "", ylab = "", partial = FALSE, fill = list(col = "#DFBEFF"),
line = list(col = "slateblue3"));
legend.1 <- sig.sym(p.model.2, sig.marks = "text", draw.always = TRUE);
legend.2 <- sig.sym(p.GT.multi.21 , sig.marks = "text", draw.always = TRUE);
legend("topright", legend = paste(legend.1, "\n", legend.2, sep = ""), bty = "n", cex =
0.9);
title(ylab="SPINA-GT (pmol/s)", line=3.3, cex.lab=1.0)

visreg(model.3, xlab = "", ylab = "", partial = FALSE, fill = list(col =
"lightskyblue2"), line = list(col = "dodgerblue3"));
legend.1 <- sig.sym(p.model.3, sig.marks = "text", draw.always = TRUE);
legend.2 <- sig.sym(p.FT4.multi.21 , sig.marks = "text", draw.always = TRUE);
legend("topleft", legend = paste(legend.1, "\n", legend.2, sep = ""), bty = "n", cex =
0.9);
title(ylab="FT4 (pmol/L)", line=3.3, cex.lab=1.0)

visreg(model.4, xlab = "", ylab = "", partial = FALSE, fill = list(col =
"paleturquoise"), line = list(col = "darkcyan"));
legend.1 <- sig.sym(p.model.4, sig.marks = "text", draw.always = TRUE);
legend.2 <- sig.sym(p.GD.multi.21 , sig.marks = "text", draw.always = TRUE);
legend("topleft", legend = paste(legend.1, "\n", legend.2, sep = ""), bty = "n", cex =
0.9);
title(ylab="SPINA-GD (nmol/s)", line=3.3, cex.lab=1.0)

visreg(model.5, xlab = "SIQALS 2", ylab = "", partial = FALSE, fill = list(col =
"#EAFF90"), line = list(col = "olivedrab4"));
legend.1 <- sig.sym(p.model.5, sig.marks = "text", draw.always = TRUE);
legend.2 <- sig.sym(p.FT3.multi.21 , sig.marks = "text", draw.always = TRUE);
legend("topleft", legend = paste(legend.1, "\n", legend.2, sep = ""), bty = "n", cex =
0.9);
title(ylab="FT3 (pmol/L)", line=3.3, cex.lab=1.0)

visreg(model.6, xlab = "SIQALS 2", ylab = "", partial = FALSE, fill = list(col =
"rosybrown1"), line = list(col = "rosybrown4"));
legend.1 <- sig.sym(p.model.6, sig.marks = "text", draw.always = TRUE);
legend.2 <- sig.sym(p.JTI.multi.21 , sig.marks = "text", draw.always = TRUE);
legend("topleft", legend = paste(legend.1, "\n", legend.2, sep = ""), bty = "n", cex =
0.9);
title(ylab="JTI", line=3.3, cex.lab=1.0)

the.plot <- annotated.barplot(heights = LBDTSH1S, categories = as.factor(SIQALS.2),
xlab = "SIQALS 2", ylab = "TSH (mIU/L)", main = "", sub = "", col = "mistyrose2",
border = "tomato3", whisk.col = "tomato3", names.arg = levels(as.factor(SIQALS.2)));
legend("topleft", legend = sig.sym(summary(model.1)$coefficients[2, 4], sig.marks =
"text", draw.always = TRUE), bty = "n")
```

```
the.plot <- annotated.barplot(heights = SPINA_GT, categories = as.factor(SIQALS.2),
xlab = "SIQALS 2", ylab = "SPINA-GT (pmol/s)", main = "", sub = "", col = "#DFBEFF",
border = "slateblue3", whisk.col = "slateblue3", names.arg =
levels(as.factor(SIQALS.2)));
legend("topleft", legend = sig.sym(summary(model.2)$coefficients[2, 4], sig.marks =
"text", draw.always = TRUE), bty = "n")

the.plot <- annotated.barplot(heights = LBDT4FSI, categories = as.factor(SIQALS.2),
xlab = "SIQALS 2", ylab = "FT4 (pmol/L)", main = "", sub = "", col = "lightskyblue2",
border = "dodgerblue3", whisk.col = "dodgerblue3", names.arg =
levels(as.factor(SIQALS.2)));
legend("topleft", legend = sig.sym(summary(model.3)$coefficients[2, 4], sig.marks =
"text", draw.always = TRUE), bty = "n")

the.plot <- annotated.barplot(heights = SPINA_GD, categories = as.factor(SIQALS.2),
xlab = "SIQALS 2", ylab = "SPINA-GD (nmol/s)", main = "", sub = "", col =
"paleturquoise", border = "darkcyan", whisk.col = "darkcyan", names.arg =
levels(as.factor(SIQALS.2)));
legend("topleft", legend = sig.sym(summary(model.4)$coefficients[2, 4], sig.marks =
"text", draw.always = TRUE), bty = "n")

the.plot <- annotated.barplot(heights = LBDT3FSI, categories = as.factor(SIQALS.2),
xlab = "SIQALS 2", ylab = "FT3 (pmol/L)", main = "", sub = "", col = "#EAFF90", border
= "olivedrab4", whisk.col = "olivedrab4", names.arg = levels(as.factor(SIQALS.2)));
legend("topleft", legend = sig.sym(summary(model.5)$coefficients[2, 4], sig.marks =
"text", draw.always = TRUE), bty = "n")

the.plot <- annotated.barplot(heights = TSHI, categories = as.factor(SIQALS.2), xlab =
"SIQALS 2", ylab = "JTI", main = "", sub = "", col = "rosybrown1", border =
"rosybrown4", whisk.col = "rosybrown4", names.arg = levels(as.factor(SIQALS.2)));
legend("topleft", legend = sig.sym(summary(model.6)$coefficients[2, 4], sig.marks =
"text", draw.always = TRUE), bty = "n")

par(oldpar);

detach(mat.thyroid.eu.allo);

if (output.to.file)
{
  dev.off();
  sink();
}
```
