# Supplementary material for: The Two Faces of Janus: Why Thyrotropin as a Cardiovascular Risk Factor May Be an Ambiguous Target
Source: Front Endocrinol (Lausanne). 2020 Oct 26;11:542710. doi: 10.3389/fendo.2020.542710 (PMC7649136; doi:10.3389/fendo.2020.542710)
Supplement: Supplementary file 3 [file DataSheet_3.pdf]

Supplementary Material

The two faces of Janus: Why thyrotropin as a cardiovascular risk factor may be an ambiguous target.

Johannes W. Dietrich*, Rudolf Hoermann, John E. M. Midgley, Friederike Bergen, Patrick Müller

*** Correspondence:** Corresponding Author: johannes.dietrich@ruhr-uni-bochum.de

Supplementary Code

S Script for meta-analysis on thyroid allostasis in PTSD. R version 3.6 or newer required (R Project for Statistical Computing, RRID:SCR_001905).

```
# Meta-Analysis of studies investigating the relationship between
# PTSD and thyroid function
# Version 1.0: 20200913 by JWD
# Version 1.1: 20200914 by JWD
# Authors: J. W. Dietrich, R. Hoermann, J. E. M. Midgley, F. Bergen and P. Mueller

if (!require("meta")) install.packages("meta");
library("meta");
if (!require("metafor")) install.packages("metafor");
library("metafor");
library("grid");

output.to.file <- TRUE;

if (Sys.info()[["sysname"]] == "Darwin" | Sys.info()[["machine"]] == "Macintosh")
{ # find the path of this script (macOS only)
  this.frame <- sys.frame(sys.nframe());
  this.dir <- dirname(sys.frame(1)$ofile);
  the.path <- paste(this.dir, "/", sep = "");
} else
  the.path <- getwd();

sig.labels <- function(p.value, sig.marks = "symbols", draw.always = FALSE)
{
  sig.label <- "";
  if ((p.value < 0.05) | draw.always)
  {
    if (sig.marks == "symbols")
      sig.label <- (if (p.value < 0.00001) '*****' else if (p.value < 0.0001) '****'
else if (p.value < 0.001) '***' else if (p.value < 0.01) '**' else if (p.value < 0.05)
'*' else 'n.s.')
    else if (sig.marks == "text")
      sig.label <- (if (p.value < 0.00001) 'p < 0.00001' else if (p.value < 0.0001) 'p
< 0.0001' else if (p.value < 0.001) 'p < 0.001' else if (p.value < 0.01) 'p < 0.01'
else if (p.value < 0.05) 'p < 0.05' else 'n.s.');
  }
return(sig.label);
}

file.tsh <- paste(the.path, "Meta-TSH.csv", sep = "");
```

```
file.ft4 <- paste(the.path, "Meta-FT4.csv", sep = "");
file.ft3 <- paste(the.path, "Meta-FT3.csv", sep = "");
file.tt4 <- paste(the.path, "Meta-TT4.csv", sep = "");
file.tt3 <- paste(the.path, "Meta-TT3.csv", sep = "");

forest.file <- paste(the.path, "forest.pdf", sep = "");
addtnl.file <- paste(the.path, "additional.pdf", sep = "");
log.file <- paste(the.path, "log.txt", sep = "");

mat.tsh.all <- read.csv2(file.tsh);
mat.tsh.inc <- mat.tsh.all[-which(mat.tsh.all$Min..Interval <= 1),]; # current criteria
mat.tsh.chronic <- mat.tsh.inc[-which(mat.tsh.inc$Min..Interval < 24),]; # chronic PTSD
only

mat.ft4.all <- read.csv2(file.ft4);
mat.ft4.inc <- mat.ft4.all[-which(mat.ft4.all$Min..Interval <= 1),]; # current criteria
mat.ft4.chronic <- mat.ft4.inc[-which(mat.ft4.inc$Min..Interval < 24),]; # chronic PTSD
only

mat.ft3.all <- read.csv2(file.ft3);
mat.ft3.inc <- mat.ft3.all[-which(mat.ft3.all$Min..Interval <= 1),]; # current criteria
mat.ft3.chronic <- mat.ft3.inc[-which(mat.ft3.inc$Min..Interval < 24),]; # chronic PTSD
only

mat.tt4.all <- read.csv2(file.tt4);
mat.tt4.inc <- mat.tt4.all[-which(mat.tt4.all$Min..Interval <= 1),]; # current criteria
mat.tt4.chronic <- mat.tt4.inc[-which(mat.tt4.inc$Min..Interval < 24),]; # chronic PTSD
only

mat.tt3.all <- read.csv2(file.tt3);
mat.tt3.inc <- mat.tt3.all[-which(mat.tt3.all$Min..Interval <= 1),]; # current criteria
mat.tt3.chronic <- mat.tt3.inc[-which(mat.tt3.inc$Min..Interval < 24),]; # chronic PTSD
only

if (output.to.file)
{
  pdf(forest.file, width = 13);
  sink(log.file, split = TRUE);
}

m.tsh.inc <- metacont(Ne, Me, Se, Nc, Mc, Sc, studlab = paste(author, year), data =
mat.tsh.inc);
m.tsh.chronic <- metacont(Ne, Me, Se, Nc, Mc, Sc, studlab = paste(author, year), data =
mat.tsh.chronic);
mr.tsh <- metareg(m.tsh.inc, Min..Interval);

m.ft4.inc <- metacont(Ne, Me, Se, Nc, Mc, Sc, studlab = paste(author, year), data =
mat.ft4.inc);
m.ft4.chronic <- metacont(Ne, Me, Se, Nc, Mc, Sc, studlab = paste(author, year), data =
mat.ft4.chronic);
mr.ft4 <- metareg(m.ft4.inc, Min..Interval);

m.ft3.inc <- metacont(Ne, Me, Se, Nc, Mc, Sc, studlab = paste(author, year), data =
mat.ft3.inc);
m.ft3.chronic <- metacont(Ne, Me, Se, Nc, Mc, Sc, studlab = paste(author, year), data =
mat.ft3.chronic);
mr.ft3 <- metareg(m.ft3.inc, Min..Interval);

m.tt4.inc <- metacont(Ne, Me, Se, Nc, Mc, Sc, studlab = paste(author, year), data =
mat.tt4.inc);
m.tt4.chronic <- metacont(Ne, Me, Se, Nc, Mc, Sc, studlab = paste(author, year), data =
mat.tt4.chronic);
mr.tt4 <- metareg(m.tt4.inc, Min..Interval);

m.tt3.inc <- metacont(Ne, Me, Se, Nc, Mc, Sc, studlab = paste(author, year), data =
mat.tt3.inc);
m.tt3.chronic <- metacont(Ne, Me, Se, Nc, Mc, Sc, studlab = paste(author, year), data =
mat.tt3.chronic);
mr.tt3 <- metareg(m.tt3.inc, Min..Interval);
```

```
cat("\nPart 1: All studies:\n");
cat("--------------------\n");
cat("\nTSH:\n");
print(m.tsh.inc);
cat("\nFT4:\n");
print(m.ft4.inc);
cat("\nFT3:\n");
print(m.ft3.inc);
cat("\nTT4:\n");
print(m.tt4.inc);
cat("\nTT3:\n");
print(m.tt3.inc);

cat("\n\n");
cat("Part 2: Studies with chronic PTSD (>= 2 years) only:\n");
cat("----------------------------------------------------\n");
cat("\nTSH:\n");
print(m.tsh.chronic);
cat("\nFT4:\n");
print(m.ft4.chronic);
cat("\nFT3:\n");
print(m.ft3.chronic);
cat("\nTT4:\n");
print(m.tt4.chronic);
cat("\nTT3:\n");
print(m.tt3.chronic);

cat("\n\n");
cat("Meta-regression:\n");
cat("----------------\n");
cat("\nTSH:\n");
print(mr.tsh);
cat("\nFT4:\n");
print(mr.ft4);
cat("\nFT3:\n");
print(mr.ft3);
cat("\nTT4:\n");
print(mr.tt4);
cat("\nTT3:\n");
print(mr.tt3);

forest(m.tsh.inc, xlab = "Difference in TSH concentration (mIU/L)", lab.e = "PTSD",
lab.c = "Control",  sortvar = author, comb.fixed = FALSE);
grid.text("Acute and chronic PTSD", x = 0.5, y = 0.95, gp=gpar(cex=1));
forest(m.tsh.chronic, xlab = "Difference in TSH concentration (mIU/L)", lab.e = "PTSD",
lab.c = "Control",  sortvar = author, comb.fixed = FALSE);
grid.text("Chronic PTSD", x = 0.5, y = 0.95, gp=gpar(cex=1));

forest(m.ft4.inc, xlab = "Difference in FT4 concentration (pmol/L)", lab.e = "PTSD",
lab.c = "Control",  sortvar = author, comb.fixed = FALSE);
grid.text("Acute and chronic PTSD", x = 0.5, y = 0.95, gp=gpar(cex=1));
forest(m.ft4.chronic, xlab = "Difference in FT4 concentration (pmol/L)", lab.e =
"PTSD", lab.c = "Control",  sortvar = author, comb.fixed = FALSE);
grid.text("Chronic PTSD", x = 0.5, y = 0.95, gp=gpar(cex=1));

forest(m.ft3.inc, xlab = "Difference in FT3 concentration (pmol/L)", lab.e = "PTSD",
lab.c = "Control",  sortvar = author, comb.fixed = FALSE);
grid.text("Acute and chronic PTSD", x = 0.5, y = 0.95, gp=gpar(cex=1));
forest(m.ft3.chronic, xlab = "Difference in FT3 concentration (pmol/L)", lab.e =
"PTSD", lab.c = "Control",  sortvar = author, comb.fixed = FALSE);
grid.text("Chronic PTSD", x = 0.5, y = 0.95, gp=gpar(cex=1));

forest(m.tt4.inc, xlab = "Difference in TT4 concentration (nmol/L)", lab.e = "PTSD",
lab.c = "Control",  sortvar = author, comb.fixed = FALSE);
grid.text("Acute and chronic PTSD", x = 0.5, y = 0.95, gp=gpar(cex=1));
forest(m.tt4.chronic, xlab = "Difference in TT4 concentration (nmol/L)", lab.e =
"PTSD", lab.c = "Control",  sortvar = author, comb.fixed = FALSE);
grid.text("Chronic PTSD", x = 0.5, y = 0.95, gp=gpar(cex=1));
```

```
forest(m.tt3.inc, xlab = "Difference in TT3 concentration (nmol/L)", lab.e = "PTSD",
lab.c = "Control",  sortvar = author, comb.fixed = FALSE);
grid.text("Acute and chronic PTSD", x = 0.5, y = 0.95, gp=gpar(cex=1));
forest(m.tt3.chronic, xlab = "Difference in TT3 concentration (nmol/L)", lab.e =
"PTSD", lab.c = "Control",  sortvar = author, comb.fixed = FALSE);
grid.text("Chronic PTSD", x = 0.5, y = 0.95, gp=gpar(cex=1));

if (output.to.file)
{
  dev.off();
  pdf(addtnl.file);
}

bubble(mr.tsh, main = "TSH concentration");
text(x = 520, y = -0.3, labels = sig.labels(mr.tsh$pval[2], sig.marks = "text",
draw.always = TRUE), pos = 2);
bubble(mr.ft4, main = "FT4 concentration");
text(x = 520, y = 1.5, labels = sig.labels(mr.ft4$pval[2], sig.marks = "text",
draw.always = TRUE), pos = 2);
bubble(mr.ft3, main = "FT3 concentration");
text(x = 520, y = -0.5, labels = sig.labels(mr.ft3$pval[2], sig.marks = "text",
draw.always = TRUE), pos = 2);
bubble(mr.tt4, main = "TT4 concentration");
bubble(mr.tt3, main = "TT3 concentration");

funnel(m.tsh.inc, main = "TSH (Acute and chronic PTSD)");
funnel(m.tsh.chronic, main = "TSH (Chronic PTSD)");
funnel(m.ft4.inc, main = "FT4 (Acute and chronic PTSD)");
funnel(m.ft4.chronic, main = "FT4 (Chronic PTSD)");
funnel(m.ft3.inc, main = "FT3 (Acute and chronic PTSD)");
funnel(m.ft3.chronic, main = "FT3 (Chronic PTSD)");
funnel(m.tt4.inc, main = "TT4 (Acute and chronic PTSD)");
funnel(m.tt4.chronic, main = "TT4 (Chronic PTSD)");
funnel(m.tt3.inc, main = "TT3 (Acute and chronic PTSD)");
funnel(m.tt3.chronic, main = "TT3 (Chronic PTSD)");

cat("\nBegg and Mazumdar test for small study effects:\n");
print(metabias(m.tsh.inc, method = "rank"));
print(metabias(m.tsh.chronic, method = "rank"));
print(metabias(m.ft4.inc, method = "rank"));
print(metabias(m.ft4.chronic, method = "rank"));
print(metabias(m.ft3.inc, method = "rank"));
print(metabias(m.ft3.chronic, method = "rank"));
print(metabias(m.tt4.inc, method = "rank"));
print(metabias(m.tt4.chronic, method = "rank"));
print(metabias(m.tt3.inc, method = "rank"));
print(metabias(m.tt3.chronic, method = "rank"));
cat("\nEgger's test for small study effects:\n");
print(metabias(m.tsh.inc, method = "linreg"));
print(metabias(m.tsh.chronic, method = "linreg"));
print(metabias(m.ft4.inc, method = "linreg"));
print(metabias(m.ft4.chronic, method = "linreg"));
print(metabias(m.ft3.inc, method = "linreg"));
print(metabias(m.ft3.chronic, method = "linreg"));
print(metabias(m.tt4.inc, method = "linreg"));
print(metabias(m.tt4.chronic, method = "linreg"));
print(metabias(m.tt3.inc, method = "linreg"));
print(metabias(m.tt3.chronic, method = "linreg"));
cat("\nThompson and Sharp test for small study effects:\n");
print(metabias(m.tsh.inc, method = "mm"));
print(metabias(m.tsh.chronic, method = "mm"));
print(metabias(m.ft4.inc, method = "mm"));
print(metabias(m.ft4.chronic, method = "mm"));
print(metabias(m.ft3.inc, method = "mm"));
print(metabias(m.ft3.chronic, method = "mm"));
print(metabias(m.tt4.inc, method = "mm"));
print(metabias(m.tt4.chronic, method = "mm"));
print(metabias(m.tt3.inc, method = "mm"));
print(metabias(m.tt3.chronic, method = "mm"));
```

```
funnel(m.tsh.inc, main = "TSH (Acute and chronic PTSD)", contour = c(0.9, 0.95, 0.99),
col.contour = c("lightgray", "gray", "darkgray"));
legend(-1.2, 0.7, c("0.1 > p >= 0.05", "0.05 > p >= 0.01", "< 0.01"), fill =
c("lightgray", "gray", "darkgray"), bty = "n");

funnel(m.tsh.chronic, main = "TSH (Chronic PTSD)", contour = c(0.9, 0.95, 0.99),
col.contour = c("lightgray", "gray", "darkgray"));
legend(0.2, 0.5, c("0.1 > p >= 0.05", "0.05 > p >= 0.01", "< 0.01"), fill =
c("lightgray", "gray", "darkgray"), bty = "n");

funnel(m.ft4.inc, main = "FT4 (Acute and chronic PTSD)", contour = c(0.9, 0.95, 0.99),
col.contour = c("lightgray", "gray", "darkgray"));
legend(-2.3, 1.4, c("0.1 > p >= 0.05", "0.05 > p >= 0.01", "< 0.01"), fill =
c("lightgray", "gray", "darkgray"), bty = "n");

funnel(m.ft4.chronic, main = "FT4 (Chronic PTSD)", contour = c(0.9, 0.95, 0.99),
col.contour = c("lightgray", "gray", "darkgray"));
legend(-1.7, 1.0, c("0.1 > p >= 0.05", "0.05 > p >= 0.01", "< 0.01"), fill =
c("lightgray", "gray", "darkgray"), bty = "n");

funnel(m.ft3.inc, main = "FT3 (Acute and chronic PTSD)", contour = c(0.9, 0.95, 0.99),
col.contour = c("lightgray", "gray", "darkgray"));
legend(-0.5, 0.27, c("0.1 > p >= 0.05", "0.05 > p >= 0.01", "< 0.01"), fill =
c("lightgray", "gray", "darkgray"), bty = "n");

funnel(m.ft3.chronic, main = "FT3 (Chronic PTSD)", contour = c(0.9, 0.95, 0.99),
col.contour = c("lightgray", "gray", "darkgray"));
legend(-0.5, 0.27, c("0.1 > p >= 0.05", "0.05 > p >= 0.01", "< 0.01"), fill =
c("lightgray", "gray", "darkgray"), bty = "n");

funnel(m.tt4.inc, main = "TT4 (Acute and chronic PTSD)", contour = c(0.9, 0.95, 0.99),
col.contour = c("lightgray", "gray", "darkgray"));
legend(50, 7, c("0.1 > p >= 0.05", "0.05 > p >= 0.01", "< 0.01"), fill = c("lightgray",
"gray", "darkgray"), bty = "n");

funnel(m.tt4.chronic, main = "TT4 (Chronic PTSD)", contour = c(0.9, 0.95, 0.99),
col.contour = c("lightgray", "gray", "darkgray"));
legend(18, 6, c("0.1 > p >= 0.05", "0.05 > p >= 0.01", "< 0.01"), fill = c("lightgray",
"gray", "darkgray"), bty = "n");

funnel(m.tt3.inc, main = "TT3 (Acute and chronic PTSD)", contour = c(0.9, 0.95, 0.99),
col.contour = c("lightgray", "gray", "darkgray"));
legend(50, 7, c("0.1 > p >= 0.05", "0.05 > p >= 0.01", "< 0.01"), fill = c("lightgray",
"gray", "darkgray"), bty = "n");

funnel(m.tt3.chronic, main = "TT3 (Chronic PTSD)", contour = c(0.9, 0.95, 0.99),
col.contour = c("lightgray", "gray", "darkgray"));
legend(18, 6, c("0.1 > p >= 0.05", "0.05 > p >= 0.01", "< 0.01"), fill = c("lightgray",
"gray", "darkgray"), bty = "n");

if (output.to.file)
{
  dev.off();
  sink();
}
```
