# Supplementary material for: The Two Faces of Janus: Why Thyrotropin as a Cardiovascular Risk Factor May Be an Ambiguous Target
Source: Front Endocrinol (Lausanne). 2020 Oct 26;11:542710. doi: 10.3389/fendo.2020.542710 (PMC7649136; doi:10.3389/fendo.2020.542710)
Supplement: Supplementary file 10 [file Image_1.pdf]

Supplementary Figures

The two faces of Janus: Why thyrotropin as a cardiovascular risk factor may be an ambiguous target.

Johannes W. Dietrich**, Rudolf Hoermann, John E. M. Midgley, Friederike Bergen, Patrick Müller

* **Correspondence:** Corresponding Author: johannes.dietrich@ruhr-uni-bochum.de

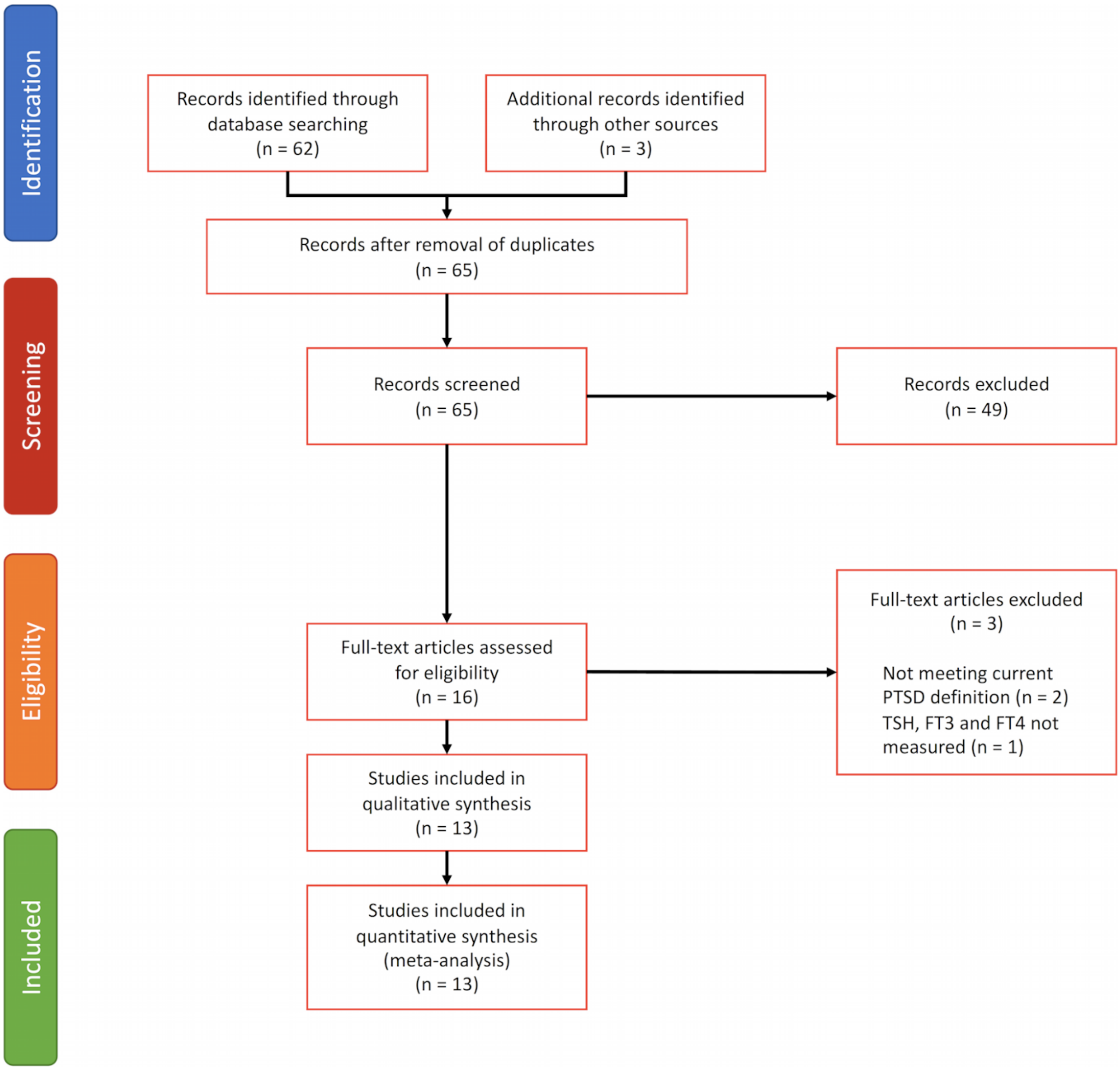

Supplementary Figure 1: PRISMA flow diagram of identified, screened and included studies

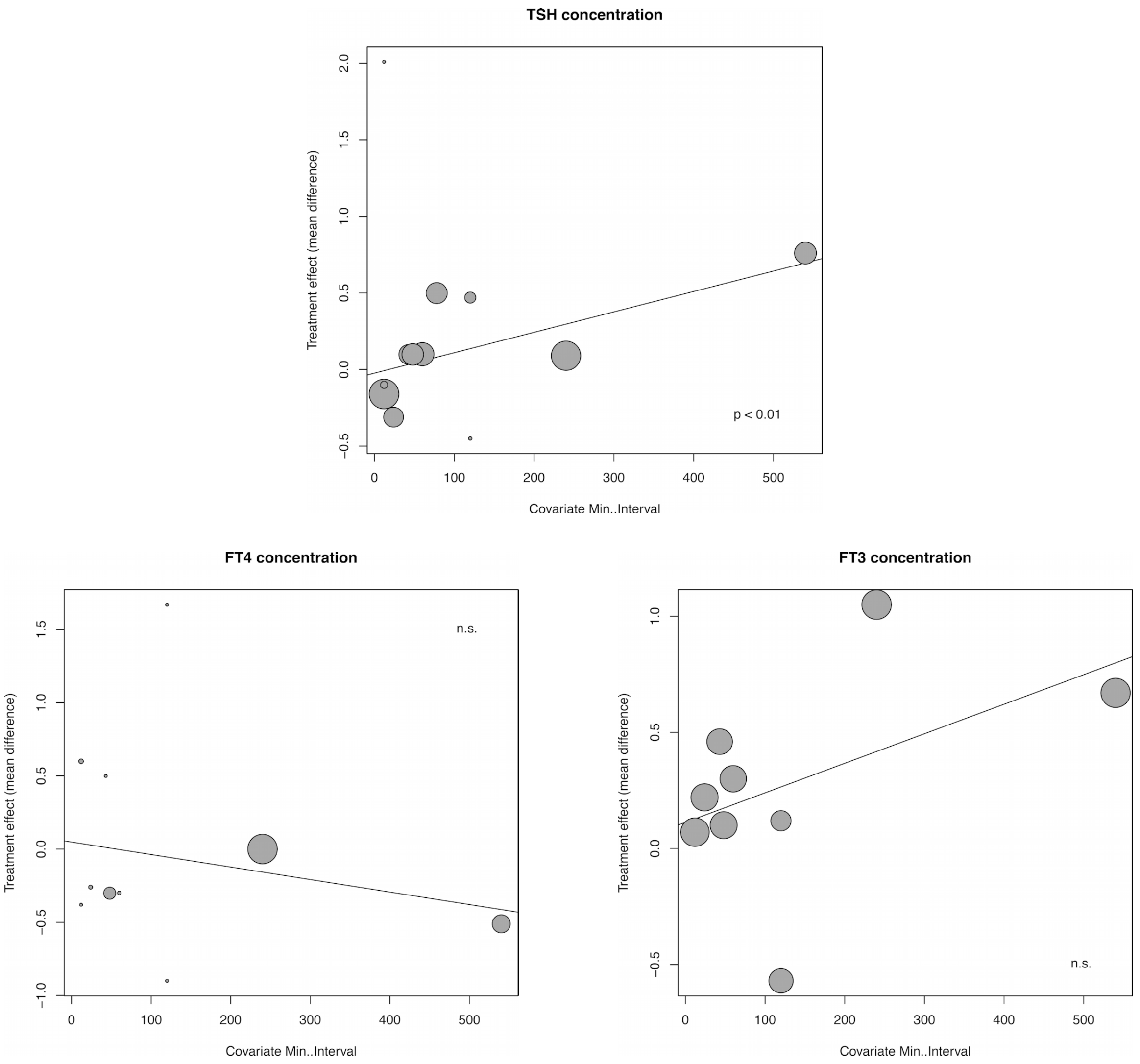

Supplementary Figure 2: Meta-regression of TSH, FT4 and FT3 concentrations with the interval between exposure to the stressor event triggering PTSD and the date of laboratory investigation of thyroid function.

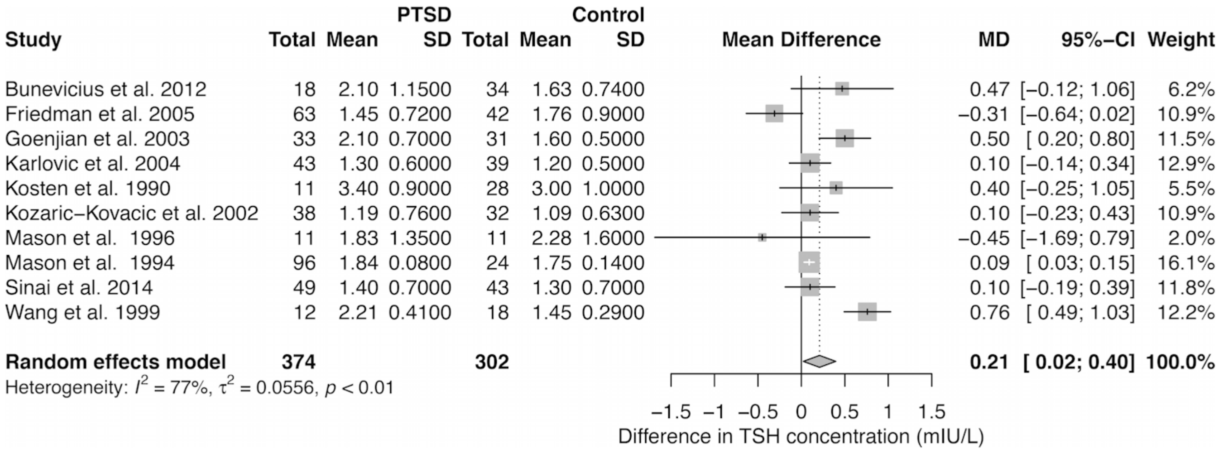

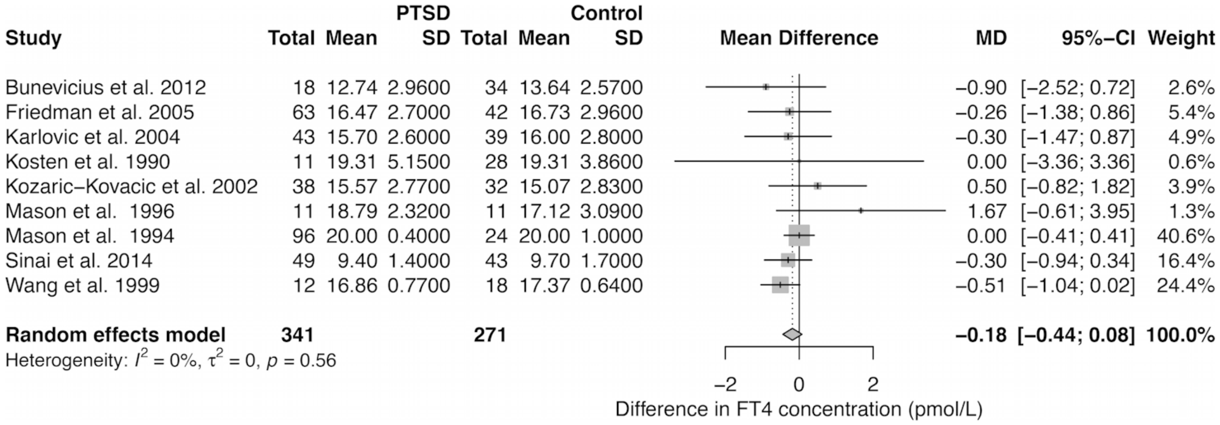

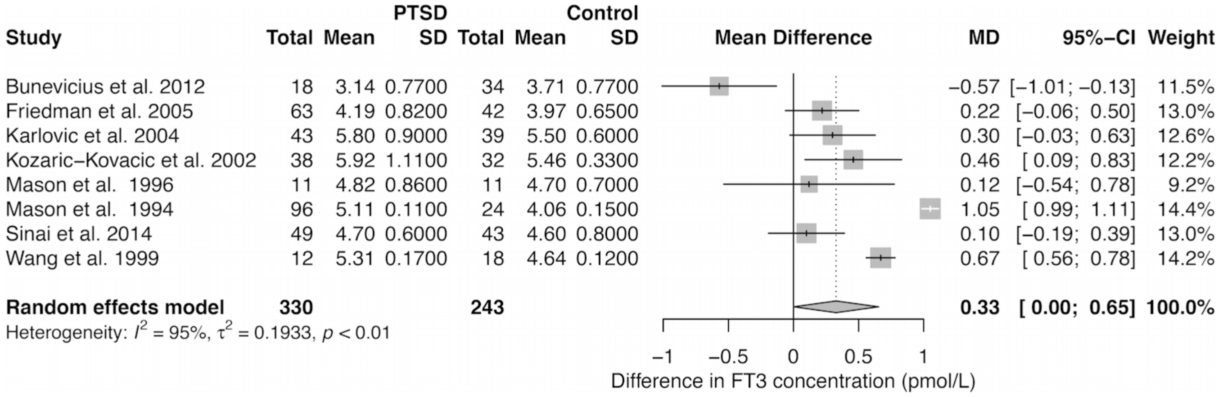

Supplementary Figure 3: Forest plots showing differences in TSH, FT4 and FT3 concentration between subjects with and without PTSD in a subset of studies with chronic PTSD (24 months or more since the occurrence of the triggering event).

Study	PTSD Total	PTSD Mean	PTSD SD	Control Total	Control Mean	Control SD	MD	95%−CI	Weight
Bunevicius et al. 2012	18	99.49	17.6300	34	96.40	15.5700	3.09	[−6.59; 12.77]	9.1%
Friedman et al. 2005	63	106.05	20.1900	42	106.05	19.1800	0.00	[−7.65; 7.65]	9.2%
Karlovic et al. 2004	43	88.20	18.3000	39	90.50	25.9000	−2.30	[−12.10; 7.50]	9.1%
Kosten et al. 1990	11	110.68	25.7400	28	100.39	16.7300	10.29	[−6.13; 26.71]	8.6%
Kozaric−Kovacic et al. 2002	38	89.00	20.1100	32	91.92	26.2000	−2.92	[−14.02; 8.18]	9.0%
Loo et al. 2013	120	194.37	19.1500	100	102.43	18.3800	91.94	[86.97; 96.91]	9.3%
Mason et al. 1996	11	121.88	15.4400	11	111.20	18.9200	10.68	[−3.75; 25.11]	8.8%
Mason et al. 1994	96	110.00	2.4000	24	87.00	3.6000	23.00	[21.48; 24.52]	9.4%
Sabioncello et al. 2000	15	123.53	16.6800	14	115.57	15.0200	7.96	[−3.58; 19.50]	9.0%
Sinai et al. 2014	45	104.00	20.0000	39	102.00	16.0000	2.00	[−5.70; 9.70]	9.2%
Wang et al. 1999	12	106.82	4.3800	18	108.11	4.8900	−1.29	[−4.64; 2.06]	9.3%
**Random effects model**	**472**			**381**			**13.11**	**[−4.17; 30.39]**	**100.0%**

Heterogeneity: $I^2 = 99\%$, $\tau^2 = 829.8283$, $p < 0.01$

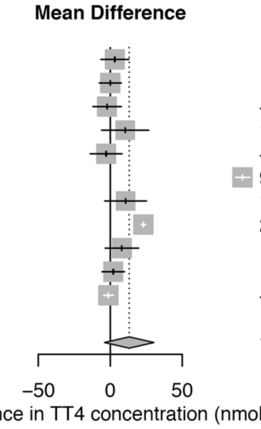

Study	PTSD Total	PTSD Mean	PTSD SD	Control Total	Control Mean	Control SD	MD	95%−CI	Weight
Bunevicius et al. 2012	18	2.02	0.3200	34	2.14	0.3300	−0.12	[−0.30; 0.06]	10.0%
Friedman et al. 2005	63	2.44	0.4100	42	2.13	0.3500	0.31	[0.16; 0.46]	10.0%
Karlovic et al. 2004	43	2.10	0.4000	39	1.70	0.4000	0.40	[0.23; 0.57]	10.0%
Kosten et al. 1990	11	110.68	25.7400	28	100.39	16.7300	10.29	[−6.13; 26.71]	0.1%
Kozaric−Kovacic et al. 2002	38	2.04	0.3200	32	1.55	0.3300	0.49	[0.34; 0.64]	10.0%
Loo et al. 2013	120	5.58	0.9700	100	1.75	0.4500	3.83	[3.64; 4.02]	10.0%
Mason et al. 1996	11	2.47	0.4200	11	2.09	0.2400	0.38	[0.09; 0.67]	9.7%
Mason et al. 1994	96	2.70	0.0600	24	2.00	0.0800	0.70	[0.67; 0.73]	10.2%
Sabioncello et al. 2000	15	2.11	0.3900	14	1.99	0.2600	0.12	[−0.12; 0.36]	9.9%
Sinai et al. 2014	43	1.90	0.3000	40	1.90	0.4000	0.00	[−0.15; 0.15]	10.0%
Wang et al. 1999	12	2.73	0.1300	18	2.34	0.0100	0.39	[0.32; 0.46]	10.1%
**Random effects model**	**470**			**382**			**0.66**	**[0.22; 1.09]**	**100.0%**

Heterogeneity: $I^2 = 99\%$, $\tau^2 = 0.4765$, $p < 0.01$

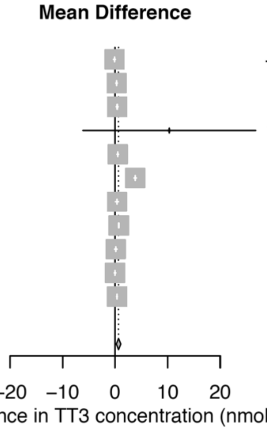

Supplementary Figure 4: Forest plots showing differences in TT4 and TT3 concentration between subjects with and without PTSD in all studies.

Study	PTSD Total	PTSD Mean	PTSD SD	Control Total	Control Mean	Control SD	MD	95%−CI	Weight
Bunevicius et al. 2012	18	99.49	17.6300	34	96.40	15.5700	3.09	[−6.59; 12.77]	10.1%
Friedman et al. 2005	63	106.05	20.1900	42	106.05	19.1800	0.00	[−7.65; 7.65]	10.4%
Karlovic et al. 2004	43	88.20	18.3000	39	90.50	25.9000	−2.30	[−12.10; 7.50]	10.0%
Kosten et al. 1990	11	110.68	25.7400	28	100.39	16.7300	10.29	[−6.13; 26.71]	8.5%
Kozaric−Kovacic et al. 2002	38	89.00	20.1100	32	91.92	26.2000	−2.92	[−14.02; 8.18]	9.8%
Mason et al. 1996	11	121.88	15.4400	11	111.20	18.9200	10.68	[−3.75; 25.11]	9.0%
Mason et al. 1994	96	110.00	2.4000	24	87.00	3.6000	23.00	[21.48; 24.52]	11.1%
Sabioncello et al. 2000	15	123.53	16.6800	14	115.57	15.0200	7.96	[−3.58; 19.50]	9.7%
Sinai et al. 2014	45	104.00	20.0000	39	102.00	16.0000	2.00	[−5.70; 9.70]	10.4%
Wang et al. 1999	12	106.82	4.3800	18	108.11	4.8900	−1.29	[−4.64; 2.06]	11.0%
**Random effects model**	**352**			**281**			**5.02**	**[−4.80; 14.84]**	**100.0%**

Heterogeneity: $I^2 = 96\%$, $\tau^2 = 225.2203$, $p < 0.01$

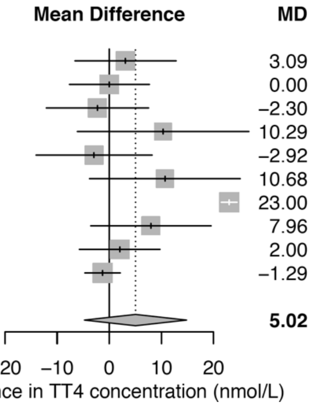

Study	PTSD Total	PTSD Mean	PTSD SD	Control Total	Control Mean	Control SD	MD	95%−CI	Weight
Bunevicius et al. 2012	18	2.02	0.3200	34	2.14	0.3300	−0.12	[−0.30; 0.06]	12.2%
Friedman et al. 2005	63	2.44	0.4100	42	2.13	0.3500	0.31	[0.16; 0.46]	12.7%
Karlovic et al. 2004	43	2.10	0.4000	39	1.70	0.4000	0.40	[0.23; 0.57]	12.4%
Kosten et al. 1990	11	110.68	25.7400	28	100.39	16.7300	10.29	[−6.13; 26.71]	0.0%
Kozaric−Kovacic et al. 2002	38	2.04	0.3200	32	1.55	0.3300	0.49	[0.34; 0.64]	12.6%
Mason et al. 1996	11	2.47	0.4200	11	2.09	0.2400	0.38	[0.09; 0.67]	10.7%
Mason et al. 1994	96	2.70	0.0600	24	2.00	0.0800	0.70	[0.67; 0.73]	13.5%
Sinai et al. 2014	43	1.90	0.3000	40	1.90	0.4000	0.00	[−0.15; 0.15]	12.6%
Wang et al. 1999	12	2.73	0.1300	18	2.34	0.0100	0.39	[0.32; 0.46]	13.3%
**Random effects model**	**335**			**268**			**0.32**	**[0.12; 0.53]**	**100.0%**

Heterogeneity: $I^2 = 96\%$, $\tau^2 = 0.0819$, $p < 0.01$

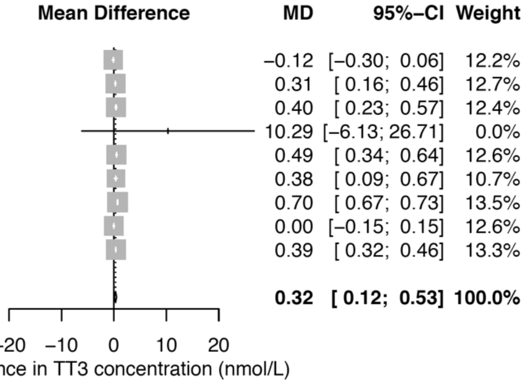

Supplementary Figure 5: Forest plots showing differences in TT4 and TT3 concentration between subjects with and without PTSD in a subset of studies with chronic PTSD.

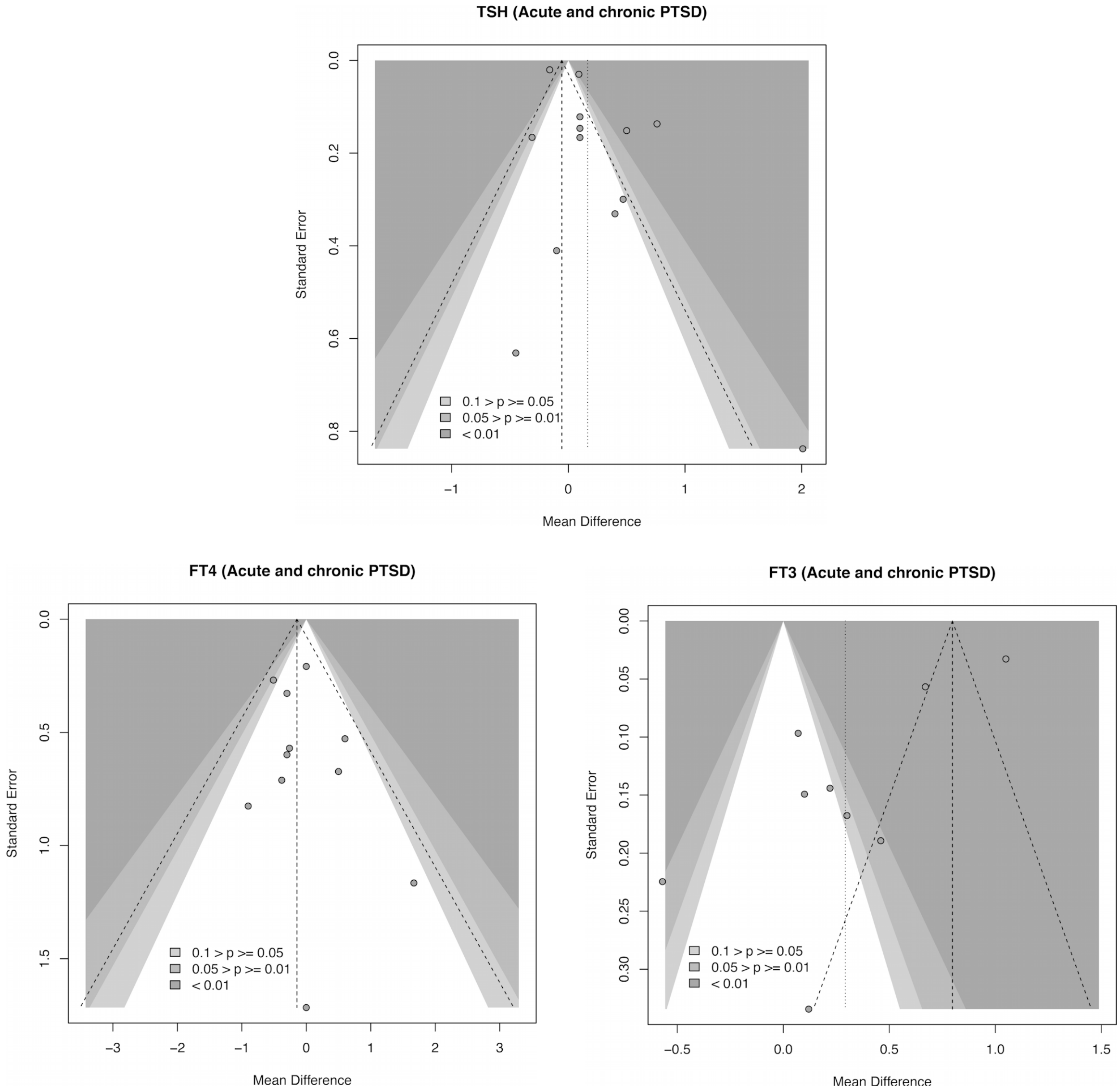

Supplementary Figure 6: Funnel plots ruling out potential small-study bias in the meta-analysis.

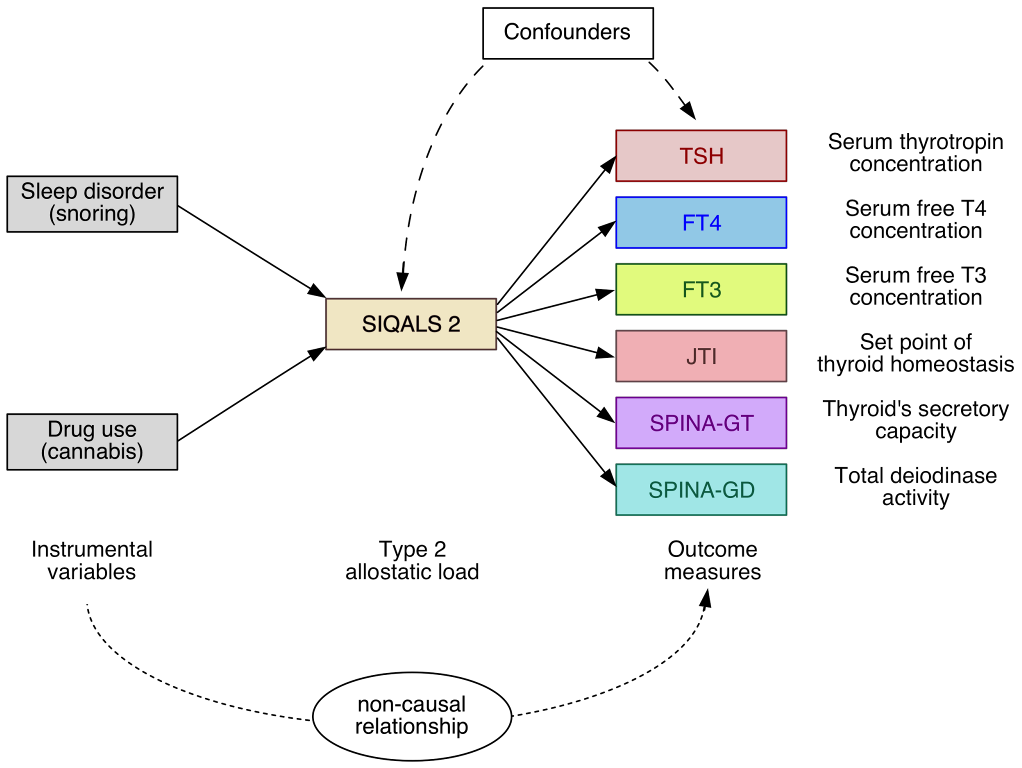

Supplementary Figure 7: Directed acyclic graph (DAG) for the used model of instrumental variable (IV) regression. Confounders are variables that influence both allostatic load and thyroid function.
