# Supplementary material for: The Two Faces of Janus: Why Thyrotropin as a Cardiovascular Risk Factor May Be an Ambiguous Target
Source: Front Endocrinol (Lausanne). 2020 Oct 26;11:542710. doi: 10.3389/fendo.2020.542710 (PMC7649136; doi:10.3389/fendo.2020.542710)
Supplement: Supplementary file 11 [file Table_1.pdf]

Supplementary tables accompanying the publication "The two faces of Janus: Why thyrotropin as a cardiovascular risk factor may be an ambiguous target."

Johannes W. Dietrich, Rudolf Hoermann, John E. M. Midgley, Friederike Bergen, Patrick Müller*

*** Correspondence:** Corresponding Author: johannes.dietrich@ruhr-uni-bochum.de

Supplementary table 1: Description of included studies. NOS: Newcastle-Ottawa score (1-13)

Study	Stressor event	Population	Design	N	NOS
**Goenjian et al. 2003**	Earthquake	Armenian adolescents	Cross-section	64	9
**Loo et al. 2013**	Cancer	Chinese women with and without breast cancer	Cross-section	220	7
**Karlovic et al. 2004**	Combat	Croatian male soldiers	Cross-section	82	7
**Kozaric-Kovacic et al. 2002**	Combat	Croatian male veterans and healthy volunteers	Cross-section	70	9
**Mason et al. 1996**	Combat	Israeli soldiers and controls	Cross-section	22	9
**Kamoi et al. 2006**	Earthquake	Japanese subjects with euthyroid Graves' disease	Prospective	69	8
**Olff et al. 2006**	Heterogeneous	Dutch outpatients with PTSD and healthy controls	Cross-section	83	8
**Bunevicius et al. 2012**	Sexual abuse	US women with and without history of sexual abuse	Cross-section	52	8
**Friedman et al. 2005**	Sexual abuse	US women with and without childhood sexual abuse	Cross-section	105	5
**Mason et al. 1994**	Combat	US combat veterans and healthy controls	Cross-section	120	7
**Wang et al. 1999**	Combat	US WWII veterans	Cross-section	30	7
**Kosten et al. 1990**	Unspecified	US PTSD patients and controls	Cross-section	39	6
**Sinai et al. 2014**	Childhood interpersonal violence	Swedish women with borderline personality disorder	Cross-section	92	6

Supplementary table 2: Raw ordinary least square (OLS) correlations between type 2 allostatic load (SIQALS 2) and parameters of thyroid homeostasis.

	B	SE	t	d.f.	p
**TSH**	0.06	0.01	5.74	3384	1.01e–8
**FT4**	0.01	0.02	0.74	3384	0.46
**FT3**	0.01	0.01	1.73	3382	0.08
**JTI**	0.04	0.01	5.84	3384	5.82e–9
**SPINA-GT**	–0.05	0.01	–4,33	3384	1.56e–5
**SPINA-GD**	0.09	0.09	1.04	3382	0.30

Supplementary table 3: Raw OLS correlations between candidates for instrumental variables and SIQALS 2.

	B	SE	t	d.f.	p
**Education**	–0.16	0.02	–7.39	3384	1.84e–13
**Poor socio-economic status**	–0.06	0.02	–3.45	2993	0.0006
**Sleep disorder (snoring)**	0.35	0.02	15.52	3140	<2e–16
**Anxiety**	0.01	0.00	3.72	3158	2e–4
**Drug use (cannabis)**	8.36e–5	1.0e–5	8.21	1370	5.13e–16

Supplementary table 4: Raw OLS correlations between candidates for instrumental variables and Jostel's TSH index (JTI).

	B	SE	t	d.f.	p
**Education**	0.01	0.01	1.46	3384	0.14
**Poor socio-economic status**	0.02	0.01	3.06	2993	0.002
**Sleep disorder (snoring)**	0.02	0.01	2.16	3140	0.03
**Anxiety**	–0.00	0.00	–0.11	3158	0.92
**Drug use (cannabis)**	1.53e–5	3.95e–6	3.87	1370	0.0001

Supplementary table 5: Raw OLS correlations between selected instrumental variables and FT4 concentration.

	B	SE	t	d.f.	p
**Poor socio-economic status**	–0.01	0.02	–0.62	2993	0.54
**Sleep disorder (snoring)**	0.02	0.02	0.86	3140	0.39
**Drug use (cannabis)**	–1.32e–5	0.99e–5	–1.33	1370	0.19

Supplementary table 6: Instrumental variables (IV) correlations between SIQALS 2 and parameters of thyroid homeostasis, based on snoring and cannabis use as instruments.

	B	SE	t	d.f.	p
**TSH**	0.13	0.05	2.60	1288	0.009
**FT4**	–0.01	0.08	–0.16	1288	0.87
**FT3**	0.01	0.03	0.24	1286	0.81
**JTI**	0.09	0.03	2.93	1288	0.003
**SPINA-GT**	–0.16	0.06	–2.77	1288	0.006
**SPINA-GD**	0.15	0.44	0.35	1286	0.73

Supplementary table 7: Variable names in the NHANES 2007–2008 dataset (14)

Concept	Variable name
**Age**	RIDAGEYR
**Sex**	RIAGENDR
**BMI**	BMXBMI
**Pulse rate**	BPXPLS
**Systolic blood pressure**	BPXSYM
**Diastolic blood pressure**	BPXDIM
**Total cholesterol concentration**	LBDTCSI
**HDL concentration**	LBDHDDSI
**HbA1c fraction**	LBXGH
**C-reactive protein concentration**	LBXCRP
**Education (highest grade of school or highest degree)**	DMDEDUC2
**Poor socio-economic status (family monthly poverty level index)**	INDFMMPI
**Sleep disorder (frequency of snoring)**	SLQ030
**Anxiety (days feeling anxious during past 30 days)**	HSQ496
**Drug use (cannabis, i.e. history of marijuana or hashish use)**	DUQ220Q
**TSH concentration**	LBXTSH1
**FT4 concentration**	LBDT4FSI
**FT3 concentration**	LBXT3F * 1.54

Supplementary References

1. Kosten TR, Wahby V, Giller E, Jr., Mason J. The dexamethasone suppression test and thyrotropin-releasing hormone stimulation test in posttraumatic stress disorder. *Biol Psychiatry* (1990) 28(8):657-64. Epub 1990/10/15. doi: 10.1016/0006-3223(90)90452-8. PubMed PMID: 2122916.
2. Mason J, Southwick S, Yehuda R, Wang S, Riney S, Bremner D, et al. Elevation of serum free triiodothyronine, total triiodothyronine, thyroxine-binding globulin, and total thyroxine levels in combat-related posttraumatic stress disorder. *Arch Gen Psychiatry* (1994) 51(8):629-41. Epub 1994/08/01. doi: 10.1001/archpsyc.1994.03950080041006. PubMed PMID: 8042912.
3. Mason J, Weizman R, Laor N, Wang S, Schujovitsky A, Abramovitz-Schneider P, et al. Serum triiodothyronine elevation with posttraumatic stress disorder: a cross-cultural study. *Biol Psychiatry* (1996) 39(10):835-8. Epub 1996/05/15. doi: 10.1016/0006-3223(95)00310-x. PubMed PMID: 9172703.
4. Wang S, Mason J. Elevations of serum T3 levels and their association with symptoms in World War II veterans with combat-related posttraumatic stress disorder: replication of findings in Vietnam combat veterans. *Psychosom Med* (1999) 61(2):131-8. Epub 1999/04/16. doi: 10.1097/00006842-199903000-00001. PubMed PMID: 10204962.
5. Kozaric-Kovacic D, Karlovic D, Kocijan-Hercigonja D. Elevation of serum total triiodothironine and free triiodothironine in Croatian veterans with combat-related post-traumatic stress disorder. *Mil Med* (2002) 167(10):846-9. Epub 2002/10/24. PubMed PMID: 12392253.
6. Goenjian AK, Pynoos RS, Steinberg AM, Endres D, Abraham K, Geffner ME, et al. Hypothalamic-pituitary-adrenal activity among Armenian adolescents with PTSD symptoms. *J Trauma Stress* (2003) 16(4):319-23. Epub 2003/08/05. doi: 10.1023/A:1024453632458. PubMed PMID: 12895013.
7. Karlovic D, Marusic S, Martinac M. Increase of serum triiodothyronine concentration in soldiers with combat-related chronic post-traumatic stress disorder with or without

alcohol dependence. *Wien Klin Wochenschr* (2004) 116(11-12):385-90. Epub 2004/08/05. doi: 10.1007/BF03040918. PubMed PMID: 15291291.
8. Friedman MJ, Wang S, Jalowiec JE, McHugo GJ, McDonagh-Coyle A. Thyroid hormone alterations among women with posttraumatic stress disorder due to childhood sexual abuse. *Biol Psychiatry* (2005) 57(10):1186-92. Epub 2005/05/04. doi: 10.1016/j.biopsych.2005.01.019. PubMed PMID: 15866559.
9. Kamoi K, Tanaka M, Ikarashi T, Miyakoshi M. Effect of the 2004 mid niigata prefecture earthquake on patients with endocrine disorders. *Endocr J* (2006) 53(4):511-21. Epub 2006/07/11. doi: 10.1507/endocrj.k06-022. PubMed PMID: 16829705.
10. Olff M, Guzelcan Y, de Vries GJ, Assies J, Gersons BP. HPA- and HPT-axis alterations in chronic posttraumatic stress disorder. *Psychoneuroendocrinology* (2006) 31(10):1220-30. Epub 2006/11/04. doi: 10.1016/j.psyneuen.2006.09.003. PubMed PMID: 17081699.
11. Bunevicius A, Leserman J, Girdler SS. Hypothalamic-pituitary-thyroid axis function in women with a menstrually related mood disorder: association with histories of sexual abuse. *Psychosom Med* (2012) 74(8):810-6. Epub 2012/09/25. doi: 10.1097/PSY.0b013e31826c3397. PubMed PMID: 23001392; PubMed Central PMCID: PMCPMC3465520.
12. Loo WT, Liu Q, Yip MC, Wang M, Chow LW, Cheung MN, et al. Status of oral ulcerative mucositis and biomarkers to monitor posttraumatic stress disorder effects in breast cancer patients. *Int J Biol Markers* (2013) 28(2):168-73. Epub 2013/05/28. doi: 10.5301/jbm.5000025. PubMed PMID: 23709344.
13. Sinai T, Hirvikoski T, Nordström A-L, Nordström P, Nilsonne Å, Wilczek A, et al. Hypothalamic pituitary thyroid axis and exposure to interpersonal violence in childhood among women with borderline personality disorder. *European Journal of Psychotraumatology* (2014) 5(1):23911. doi: 10.3402/ejpt.v5.23911.
14. National Center for Health Statistics. National Health and Nutrition Examination Survey: Centers for Diesase Control and Prevention (2020) [20200302]. Available from: https://www.cdc.gov/nchs/nhanes/index.htm.
